# Free-hand gas identification based on transfer function ratios without gas flow control

**DOI:** 10.1038/s41598-019-46164-1

**Published:** 2019-07-05

**Authors:** Gaku Imamura, Kota Shiba, Genki Yoshikawa, Takashi Washio

**Affiliations:** 1 World Premier International Research Center Initiative (WPI), International Center for Materials Nanoarchitectonics (MANA), National Institute for Materials Science (NIMS), Namiki 1-1, Tsukuba, Ibaraki 305-0044 Japan; 2 Center for Functional Sensor & Actuator (CFSN), National Institute for Materials Science (NIMS), Namiki 1-1, Tsukuba, Ibaraki 305-0044 Japan; 3 Materials Science and Engineering, Graduate School of Pure and Applied Science, University of Tsukuba, Tennodai 1-1-1, Tsukuba, Ibaraki 305-8571 Japan; 4 The Institute of Scientific and Industrial Research, Osaka University, Mihogaoka 8-1, Ibaraki, Osaka, 567-0047 Japan

**Keywords:** Sensors, Applied physics

## Abstract

Gas identification is one of the most important functions of a gas sensor system. To identify gas species from sensing signals without gas flow control such as pumps or mass flow controllers, it is necessary to extract decisive dynamic features from complex sensing signals due to uncontrolled airflow. For that purpose, various analysis methods using system identification techniques have been proposed, whereas a method that is not affected by a gas input pattern has been demanded to enhance the robustness of gas identification. Here we develop a novel gas identification protocol based on a transfer function ratio (TFR) that is intrinsically independent of a gas input pattern. By combining the protocol with MEMS-based sensors—Membrane-type Surface stress Sensors (MSS), we have realized gas identification with a *free-hand* measurement, in which one can simply hold a small sensor chip near samples. From sensing signals obtained through the *free-hand* measurement, we have developed highly accurate machine learning models that can identify odors of spices and herbs as well as solvent vapors. Since no bulky gas flow control units are required, this protocol will expand the applicability of gas sensors to portable electronics, leading to practical artificial olfaction.

## Introduction

Recent advances in information and communication technology (ICT) have stimulated huge demand for sensors, which play a key role in highly integrated systems—for example, cyber-physical systems (CPS) and Internet of things (IoT)^1^. Among sensors, gas sensors have been used in various applications such as detection of toxic gases, monitor of indoor air quality, and automotive emissions control^2^. In addition to these applications which basically focus on single component gases, development of a system that can detect and identify odors—complex mixture of gases—has been a long-standing issue since Persaud and Dodd published the first report on artificial olfaction based on gas sensors in 1982^3^. The basic concept of artificial olfaction is as follows: first, an odor is detected with an array of gas sensors, each of which shows a different sensing property. Then, the signal features are extracted and selected from the sensing signals. Finally, the odor is identified on the basis of the features through a classification algorithm. Therefore, it is necessary to develop both gas sensors and data analysis methods to realize artificial olfaction. Many studies on artificial olfaction have been published so far^4,5^, and some products are already commercially available. However, products that meet the requirements for consumer use have not shown up yet; odor identification devices which are low-priced, portable, and easy-to-use have not been achieved. To realize such a practical artificial olfaction, it is essential to develop a simple and compact measurement system that people without specific expertise can use.

For realizing practical artificial olfaction, gas flow control is one of the biggest problems. To obtain comparable sensing signals, it is required to employ the same gas flow sequence for every measurement^6^. Thus, in many odor measurement systems, a sample gas is injected to a gas sensor array by gas flow control units such as pumps and mass flow controllers (MFCs). Typically, a sample gas and a carrier gas are alternately injected to a gas sensor, resulting in a periodic peaks of sensing responses. From such sensing signals, features such as slope, area, and decay time are extracted for analysis. However, measurement data obtained with a different gas flow sequence cannot be compared with each other because the shape of sensing signals strongly depends on the gas flow sequence in this measurement protocol. To resolve this issue, gas identification methods based on system identification have been developed. In 1994, Nakamura *et al*. demonstrated that the sensing response of quartz crystal microbalance (QCM) can be analyzed by autoregressive (AR) models^7^, leading to the development of analysis with advanced time-series models such as autoregressive models with exogenous input (ARX) and autoregressive moving average (ARMA) models^8,9^. Analysis methods based on an impulse response function and a transfer function were also developed to describe the dynamic behavior of sensor responses to varying gas concentration^8,10,11^. Furthermore, pioneering work was done by Marco and Pardo and their coworkers to adapt non-linear models including artificial neural networks (ANN) and Wiener kernel analysis for describing the complex response of sensing systems^12–14^. In 2015, Fonollosa *et al*. reported a gas concentration estimation from randomly changing gas input pattern based on reservoir computing algorithm, which is one kind of recurrent neural network (RNN)^15^.

The most challenging measurement style is an open sampling condition, in which sensors are directly exposed to sample gases without any gas flow control. In an open sampling condition, the gas input pattern is neither controlled nor monitored. Several groups have reported gas identification in open sampling conditions. Trincavelli and his colleagues developed a gas identification system with continuous sampling, in which the system continuously intakes a plume of a sample gas^16–19^. The authors extracted features from the transient responses through curve fitting, discrete Fourier transform, and discrete wavelet transform, and identified the gas species by support vector machines, demonstrating the feasibility of a mobile robot equipped with an electronic nose for rapid odor classification. Monroy *et al*. demonstrated gas quantification from sensing signals of metal oxide (MOX) gas sensors exposed to turbulent airflow of sample gases^20^. The authors utilized Gaussian process for analyzing the sensing signals, resulting in the prediction of the gas concentration with the uncertainty of the estimate. Esposito *et al*. employed dynamic neural network (DNN) for estimating the concentration of specific gases from the sensing signals obtained with multisensory devices which were located outside^21,22^. Vergara *et al*. investigated the classification performance of gas sensor arrays by using a wind tunnel testbed facility where gas sensor arrays are exposed to sample gases, whereas the authors used the response of each gas sensor at a steady state as features for classification^23^. Related to these works, Han *et al*. developed an improved classification algorithm based on an unsupervised learning—the *KmP* algorithm^24^. Although such studies for gas identification in the open sampling condition have exploited gas identification protocol without gas flow control, an analysis method based on signal features that are *intrinsically* independent of the gas input pattern—signals features that are determined only by the combination of a sensor and a gas species—is still required. Using such intrinsic signal features, gas identification methods which are highly robust to the gas input pattern can be realized. Toward a practical application of artificial olfaction, further breakthrough is still needed to enhance the usability including robustness and portability of the measurement system.

**Figure 1:**
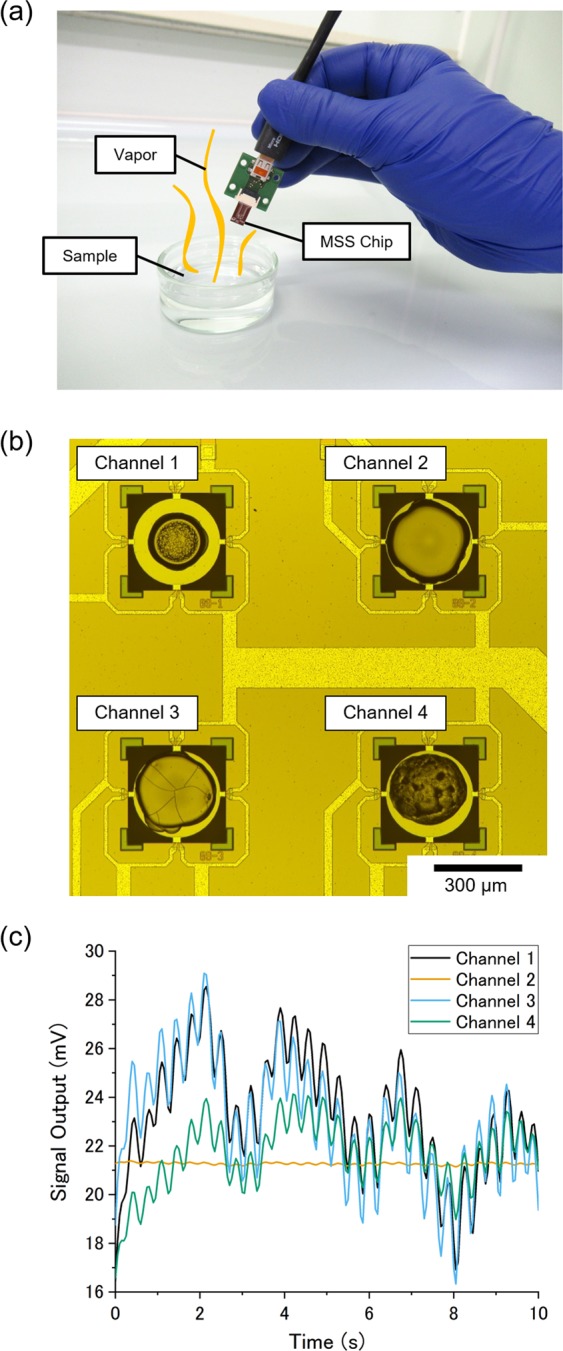
(**a**) The picture of the free-hand measurement with the illustration of sample vapor. (**b**) The optical microscope image of MSS Chip I. Channels 1 to 4 are coated with poly(vinylidene fluoride), polysulfone, poly(4-methylstyrene) and polycaprolactone, respectively. (**c**) Sensing signals of MSS Chip I measuring ethyl acetate through the free-hand measurement; manually moving the chip at roughly 3 Hz.

In this study, we have developed a novel gas identification protocol for an open sampling condition based on the transfer function ratio (TFR), which are intrinsic to gas species and independent of the gas input pattern. By focusing on the TFR, control or monitor of the gas input pattern is no longer required for gas identification because TFR is *intrinsically* independent of gas input patterns and can be calculated only from sensing signals of arrayed gas sensors as explained later in detail. Combined with a miniaturized sensor including a MEMS sensor, this gas identification protocol realizes a compact measurement system in which gas species are identified through a *free-hand measurement*—sample gases are measured with a small sensor chip by manually moving the sensor chip near the sample (Fig. 1a). To demonstrate the gas identification through the free-hand measurement, we employed Membrane-type Surface stress Sensors (MSS) as gas sensors in this study because of their high sensitivity, compactness, and wide variation in sensing properties (Fig. 1b)^25,26^. From the measurement data obtained through the free-hand measurement (Fig. 1c), we developed machine learning models for classifying gas species, resulting in identification of four solvent vapors at an accuracy of 0.996 ± 0.006. Furthermore, we applied this gas identification protocol to odors of spices and herbs, resulting in a classification accuracy of 0.89 ± 0.04. This result shows that not only single component gases but also multicomponent gas mixtures can be identified simply by moving a small sensor chip near samples. The robustness of the TFR-based analysis to gas input patterns was confirmed by developing a classification model from measurement data obtained with two different types of gas injection sequences. As shown in Supplementary Video, the TFR-based gas identification protocol combined with MSS realizes easy and rapid identification of gas species, leading to implementation of artificial olfaction in various portable devices and wearable devices.

## Proposed Gas Identification Protocol

A transfer function is one of the mathematical representations to describe a process model of a system. Assuming that a gas sensing system exhibits linear response, in which an output sensing signal *y*(*t*) is linear in the gas injection pattern *x*(*t*), *y*(*t*) can be described as a convolution of *x*(*t*) and the time-domain transfer function (or the impulse response function) *h*_*g*_(*t*):

1 $$y(t)={\int }_{0}^{t}{h}_{g}(\tau )x(t-\tau )d\tau $$

Here, *h*_*g*_(*t*) is determined by the interaction between the sensor and a gas *g*. As *h*_*g*_(*t*) does not depend on *x*(*t*), *h*_*g*_(*t*) is considered to be an intrinsic function of the gas, leading to gas identification based on *h*_*g*_(*t*). By applying the Fourier transform, the frequency-domain expression for Eq. (1) can be obtained as the following form:

2 $$Y(f)={H}_{g}(f)X(f)$$

where *X*(*f*), *Y*(*f*), and *H*_*g*_(*f*) are the frequency-domain expressions for the gas injection pattern, the output sensing signal, and the transfer function, respectively^8^.

It is, of course, possible to identify gas species by the transfer function *H*_*g*_(*f*) calculated from gas input pattern *X*(*f*) (*e*.*g*. gas flow rate or gas concentration) and sensing signals *Y*(*f*). In such a straightforward approach, however, *X*(*f*) still needs to be measured by controlling or monitoring gas injections to calculate *H*_*g*_(*f*). This issue can be solved by using an array of gas sensors with different sensing characteristics. Considering that a gas *g* is provided to an array of gas sensors according to *X*(*f*), the output sensing signal of the *i*th channel of the sensor array *Y*_*i*_(*f*) is described as the following form:

3 $${Y}_{i}(f)={H}_{g,i}(f)X(f)$$

where *H*_*g*,*i*_(*f*) is the transfer function of the *i*th channel for the gas *g*. If the gas sensor channels in the array can be considered to be spatially equivalent for the gas input, *X*(*f*) is the same for all the channels. Thus, for any combination of two channels *m* and *n*, the following equation holds:

4 $$X(f)=\frac{{Y}_{m}(f)}{{H}_{g,m}(f)}=\frac{{Y}_{n}(f)}{{H}_{g,n}(f)}$$

Let *K*_*m*,*n*_(*f*) be defined as *K*_*m*,*n*_(*f*) = *Y*_*m*_(*f*)/*Y*_*n*_(*f*), which is the signal ratio of the *m*th and *n*th channels in the frequency domain. Then, *K*_*m*,*n*_(*f*) can be described as the following form from Eq. (4):

5 $${K}_{m,n}(f)=\frac{{Y}_{m}(f)}{{Y}_{n}(f)}=\frac{{H}_{g,m}(f)}{{H}_{g,n}(f)}$$

As *H*_*g*,*m*_(*f*)/*H*_*g*,*n*_(*f*) is the TFR of the *m*th and *n*th channels, *K*_*m*,*n*_(*f*) is the *intrinsic* value to the gas *g*. Here, it is noteworthy that *K*_*m*,*n*_(*f*) is independent of *X*(*f*); that is, *K*_*m*,*n*_(*f*) can be estimated from any gas input pattern. Therefore, by calculating *K*_*m*,*n*_(*f*) from an arbitrary combination of two channels from a gas sensor array, it is possible to identify a gas species without controlling or monitoring the gas input pattern.

## Experimental Setup

On the basis of the gas identification protocol focusing on the TFR, it is possible to identify gas species with only a gas sensor array; gas flow lines including pumps or MFCs are no longer needed as long as a time-varying gas input that is consistent for all sensor channels is provided. Thus, in this study, we demonstrate gas identification through the free-hand measurement; sample gases are measured by manually moving a miniaturized gas sensor array near the samples. For this purpose, we utilized MSS as a sensing platform. An MSS is a kind of nanomechanical sensors, which detect changes in mechanical properties such as mass, stress, and deformation^27^. An MSS detects surface stress associated with gas sorption or desorption at a receptor layer. Its unique structure—a silicon membrane coated with a receptor material is suspended by four beams in which piezoresistors are embedded—effectively transduces the surface stress into electrical signals. MSS are suitable for the free-hand measurement owing to the following reasons. Firstly, the sensing elements (membranes) can be densely arrayed: more than 100 elements/cm^2^ ^28^. As the gas identification protocol assumes that all the channels in an array are spatially equivalent, gas sensors must be miniaturized so that all the channels are arrayed in a small area. Secondly, MSS realize various sensing characteristics, which are preferable to obtain unique *K*_*m*,*n*_(*f*). As MSS detect surface stress caused by gas sorption/desorption of the receptor material, almost all solid materials can be utilized as receptor materials of MSS, leading to a wide variety of sensing characteristics^29–31^. Thus, we developed an MSS-based measurement system, which allows the free-hand measurement without gas flow control units such as pumps or MFCs. From the data obtained through the free-hand measurements, machine learning models for gas identification were developed.

## Results and Discussion

### Free-hand measurement system

In this study, we used MSS chips with four channels, which were coated with different receptor materials. As receptor materials, we utilized polymers, which have been widely used in the field of nanomechanical sensors because of their preferable mechanical properties and the wide variation^32,33^. In this study, we coated the channels of an MSS chip with poly(vinylidene fluoride), polysulfone, poly(4-methylstyrene), and polycaprolactone by inkjet spotting (Fig. 1b). (hereafter, the polymer-coated MSS chip is described as “MSS Chip I”.) To demonstrate gas identification with the free-hand measurement, we measured vapors of four solvents: ethanol, water, heptane, and ethyl acetate. The gas measurements were conducted by manually moving the MSS chip in the vapor of samples as shown in Fig. 1a. We measured each sample eight times for 90 seconds each time. Figure 1c shows one example of the sensing signals obtained with MSS Chip I. Each channel shows a different sensing response (*e*.*g*. amplitude and phase shift) according to the fluctuation of gas concentration at the chip mainly associated with a motion of a hand, which moved at roughly 3 Hz.

**Figure 2:**
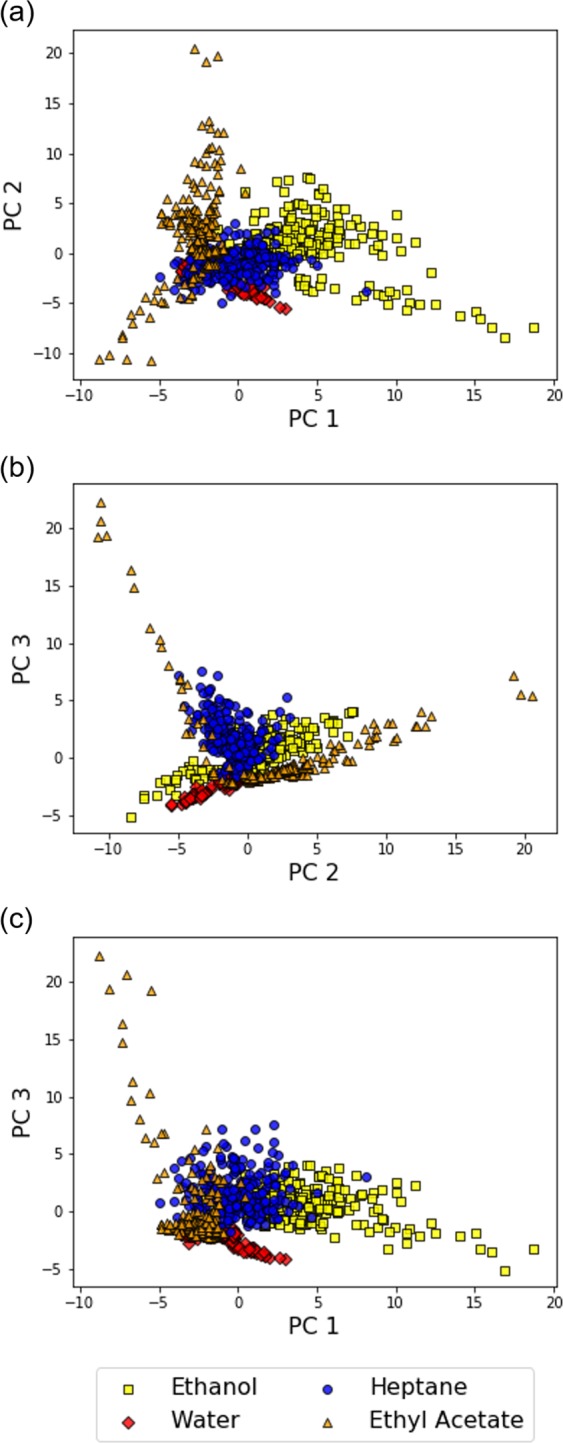
The scatter plots of PCA (**a**–**c**) on the dataset of the solvent vapors obtained with MSS Chip I. One data point represents one segmented data.

Using MSS Chip I, we measured the headspace vapors of the solvents through the free-hand measurement. From the measurement data, we calculated *K*_*m*,*n*_(*f*) and created a dataset for analysis (The plots for *K*_*m*,*n*_(*f*) obtained with MSS Chip I are shown in SI). To visualize the dataset and verify the potential of *K*_*m*,*n*_(*f*) as a feature for gas identification, we first performed a typical dimensionality reduction algorithm on the dataset: principal component analysis (PCA). PCA projects data points to a low dimensional space consisting of principal components, which are determined by the variance of the dataset. Figure 2a–c show the results of PCA. Although the gas species are not completely discriminated in the plots, the data points belonging to the same gas species form a cluster on the feature spaces. As the clusters are roughly separated from each other, *K*_*m*,*n*_(*f*) reflects the different interaction between the receptor materials and the gas species.

**Figure 3:**
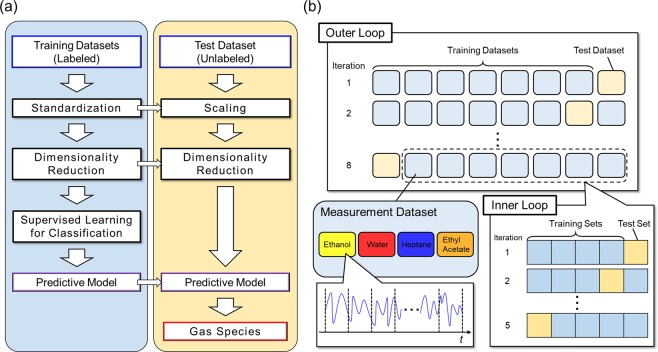
(**a**) Basic procedure for developing a classification model. (**b**) Illustration of the nested CV. A rounded rectangle in “Outer Loop” represents an individual dataset consisting of four measurement files for the vapors. Each measurement file is segmented. Training datasets in the outer loop are randomly split into five datasets (rectangles in “Inner Loop”).

As the results of PCA show the potential of TFRs as descriptors of gas species, we then developed machine learning models for identifying the solvent vapors. The basic procedure for developing machine learning models for classifying the vapors (odors) is illustrated in Fig. 3a. First, the training datasets were standardized, followed by dimensionality reduction. The datasets were not standardized when decision trees and random forests are used as classifiers. The classifier was then trained on the basis of the data with their class labels, resulting in development of a prediction model. The prediction model was validated with the test dataset. Note that the test dataset was processed with the same parameters (i.e. mean, standard deviation, and the transformation matrix) as the training datasets for scaling and dimensionality reduction. In this study, the machine learning models were developed through the nested cross validation (CV). The schematic illustration of the nested CV is shown in Fig. 3b. A rounded rectangle in “Outer Loop” represents an individual measurement dataset, which consists of four measurement files for the vapors. A prediction model was developed and optimized with the seven datasets while the developed model was evaluated with the remaining one dataset. Such a model building and evaluation process was performed for all the combinations of training and test datasets (outer loop). It should be noted that the developed prediction model was tested by measurement files which are independent of the ones used for training; the same measurement file is not shared between the training datasets and the test dataset. A prediction model was optimized through the 5-fold CV in the inner loop. The datasets in the inner loop were randomly split into training and test sets from the training datasets in the outer loop. In this study, PCA was employed for the dimensionality reduction algorithm, and the following six classification algorithms were utilized as classifiers: support vector machines (SVMs) with a linear kernel, SVMs with a radial basis function (RBF) kernel, logistic regression (LR), decision trees (DTs), random forests (RFs), and multilayer perceptrons (MLPs). The hyperparameters of the classifiers are summarized in Table 1. Both the number of principal components (*N*_*PC*_) and hyper-parameters of each classifier (e.g. the regularization parameter *C*) were optimized through the cross-validated grid-search. In the model building with logistic regression and support vector machine as classifier, models were also tested for *N*_*PC*_ = 10, 20, 40, and 80.

**Table 1:** Hyperparameters of classifiers.

Parameter	Value
SVM (Linear)	Penalty parameter *C* of the error term
SVM (RBF Kernel)	Penalty parameter *C* of the error term Kernel coefficient *γ* for the RBF
Logistic Regression	Penalty parameter *C* of the error term
Decision Tree	The maximum depth of the tree
Random Forest	The number of trees in the forest
Multilayer Perceptron	L2 penalty parameter *α* The hidden layer sizes

The results of the models are summarized in Table 2. We achieved classification accuracies of approximately 0.85 with these classifiers, particularly the classification model based on random forests exhibits the highest accuracy among the six models (0.90 ± 0.15). These results indicate that gas species could be identified with high accuracy by the free-hand measurement—simply moving the MSS chip in the vapor of the samples without any gas flow control. It should be noted that the measurement time required to identify gas species is only 3.0 seconds, demonstrating a rapid (practically real time) gas identification.

**Table 2:** Results of classification models for solvent vapors using MSS Chip I.

Classifier	Optimized Parameters	Accuracy
Support Vector Machine (Linear Kernel)	Number of PCs: 40 *C*: 0.1	0.82 ± 0.15
Support Vector Machine (RBF Kernel)	Number of PCs: 80 *C*: 10.0 *γ*: 0.01	0.83 ± 0.18
Logistic Regression	Number of PCs: 20 *C*: 10.0	0.82 ± 0.16
Decision Tree	Maximum depth: 10	0.81 ± 0.20
Random Forest	Number of estimators: 1024	0.90 ± 0.15
Multilayer Perceptron	*α*: 1.0 Hidden layer size: (128,128,64)	0.84 ± 0.16

### Optimization of receptor materials

**Figure 4:**
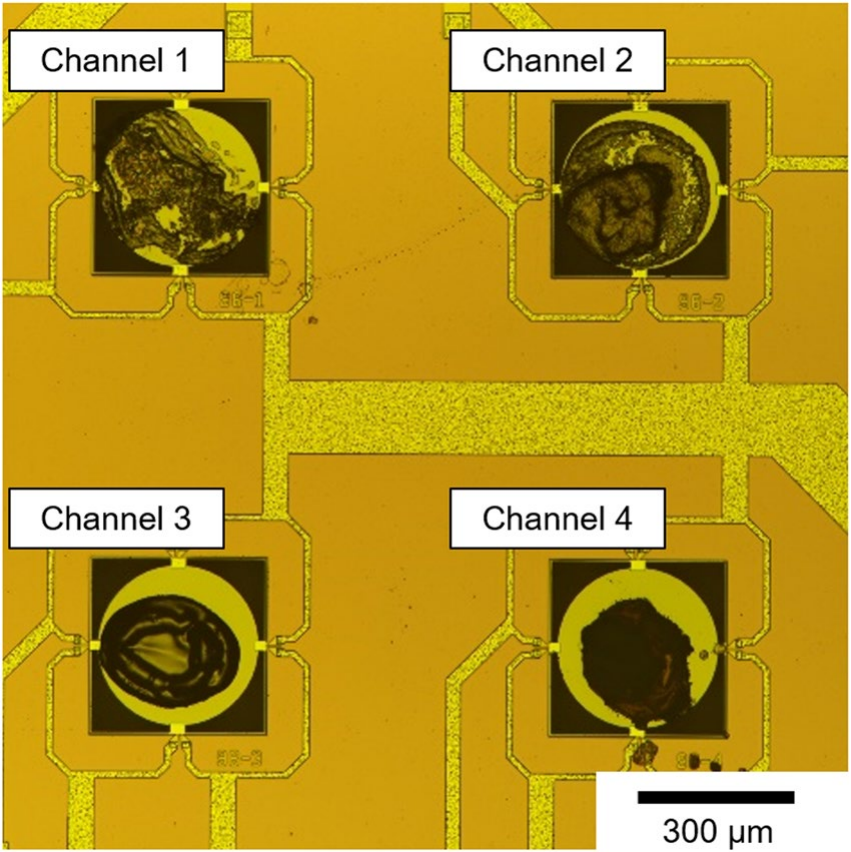
The optical microscope image of MSS Chip II. Channels 1 to 4 are coated with C18-STNPs, Ph-STNPs, NH2-STNPs, and silica-C16TA hybrid particles, respectively.

**Figure 5:**
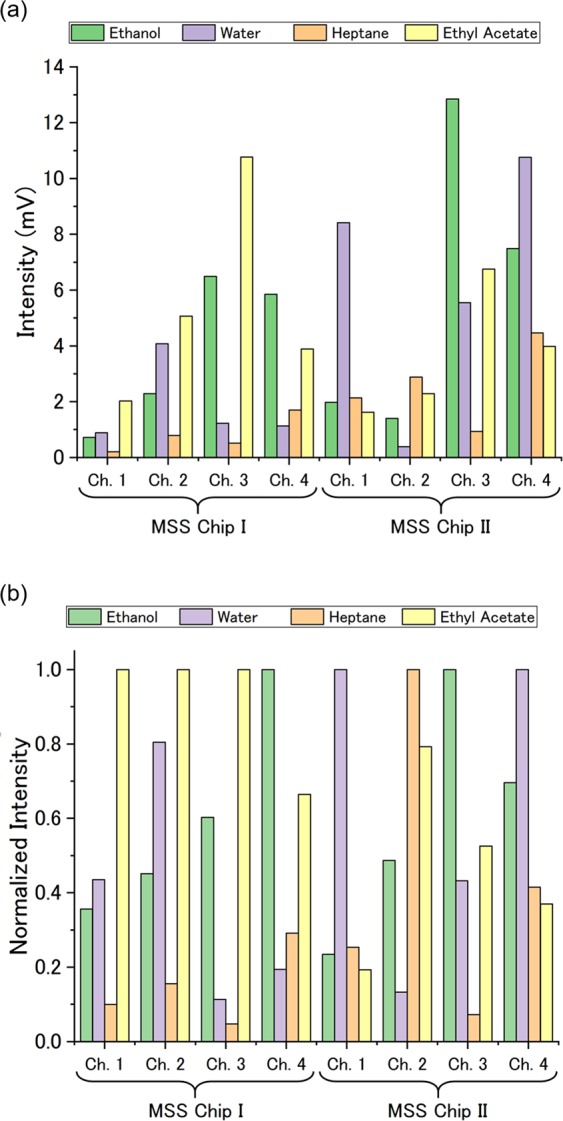
(**a**) The intensities of MSS Chip I and MSS Chip II to the solvent vapors. (**b**) The intensities normalized by the highest response for each channel.

To improve the accuracy, we optimized the receptor layers of the MSS. As an effective platform of receptor materials, we employed functional inorganic nano-/micro-particles. The nano-/micro-particles have several advantages over polymer. For example, their high surface area to volume ratio leads to high sensitivity and short response time. In the case of nanomechanical sensing, high sensitivity can be expected for inorganic nano-/micro-particles owing to their high Young’s moduli^34,35^. In addition, the surface of the inorganic nano-/micro-particles can be functionalized with various kinds of moieties, providing wide variation of chemical selectivity. To enhance the chemical selectivity without deteriorating the sensitivity compared to MSS Chip I, we utilized three kinds of functional silica/titania hybrid nanoparticles (STNPs) and one kind of hybrid particles as receptor materials: C18-STNPs, Ph-STNPs, NH2-STNPs, and silica-hexadecyltrimethylammonium hybrid particles (silica-C16TA hybrid). The STNPs were synthesized via sol-gel reaction of two alkoxides such as titanium tetraisopropoxide and silane coupling reagent^36,37^. The details of the synthesis method for these functional STNPs are described in Refs^32^ and^33^. The silica-C16TA hybrid was synthesized by the Stöber method combined with the supramolecular templating approach reported previously^38^. The STNPs were coated on the channels of an MSS chip through the same procedure as MSS Chip I. (hereafter “MSS Chip II”.) Figure 4 shows the optical microscope image of MSS Chip II. The intensities of MSS Chip I/II to the four solvent vapors are summarized in Fig. 5. As expected, MSS Chip II exhibited larger variation in the intensities to each vapor than MSS Chip I, indicating that MSS Chip II is more capable of discriminating the vapors than MSS Chip I. The quantitative analysis is described in Supplementary Information.

**Figure 6:**
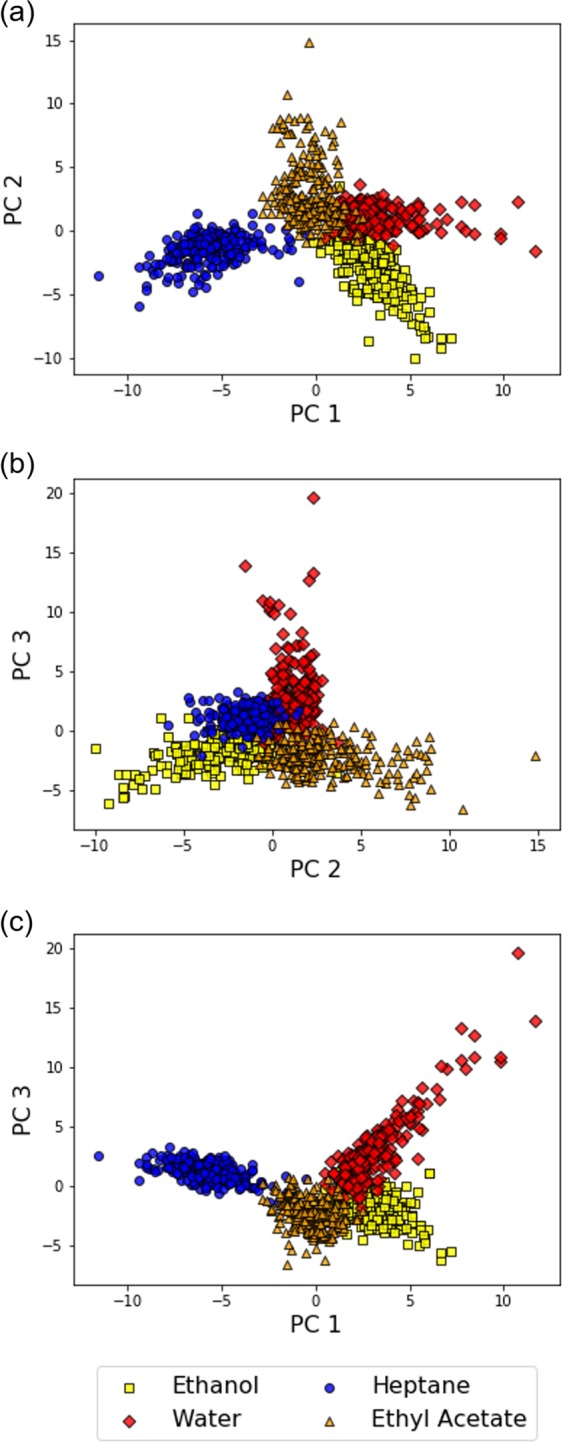
The scatter plots of PCA on the dataset of the solvent vapors obtained with MSS Chip II.

Using MSS Chip II, we conducted the free-hand measurement for the four solvent vapors. Figure 6a–c show the results of PCA from the data obtained with MSS Chip II (The plots for *K*_*m*,*n*_(*f*) obtained with MSS Chip II are shown in SI). The clusters appearing in Fig. 6 seem to be more separated from each other than those in Fig. 2. To quantitatively evaluate the cluster quality of the results in Figs 2 and 6, we calculated the Davies–Bouldin (DB) index—a common index that estimates the cluster separation—for the datasets obtained with MSS Chip I and MSS Chip II. The DB index for a dataset consisting of *n* clusters is defined as the following formula:

6 $$DB\,=\frac{1}{n}\sum _{i}^{n}\mathop{\max }\limits_{i\ne j}(\frac{{\sigma }_{i}+{\sigma }_{j}}{d({c}_{i},{c}_{j})})$$

where *c*_*k*_ and *σ*_*k *_are the centroid of cluster *k* and the mean distance of all elements in cluster *k* from *c*_*k*_, respectively. Distance between *c*_*k*_ and *c*_*l*_ is denoted as d(*c*_*k*_, *c*_*l*_). As can be inferred from Eq. (6), a DB index becomes low when clusters are well separated from each other; that is, the radius of each cluster is small, and the distance between two clusters is large. The *DB*s for the datasets obtained from MSS Chip I and MSS Chip II are 3.34 and 1.53, respectively. Therefore, the optimization of the receptor materials based on the chemical selectivity resulted in the improvement in cluster quality of the measurement dataset, leading to better gas discrimination.

The accuracy of the machine learning models was also improved by the optimization. Table 3 summarizes the details of the developed machine learning models. Compared to the previous results in Table 2, all the models developed from the data obtained with MSS Chip II show high accuracies over 0.95. Among the models, the random forest-based model achieved considerably high accuracy (0.996 ± 0.006). It is noteworthy that the standard deviation of the accuracy is decreased for MSS Chip II, indicating that measurement data with MSS Chip II are more reliable than those with MSS Chip I.

**Table 3:** Results of classification models for solvent vapors using MSS Chip II.

Classifier	Optimized Parameters	Accuracy
Support Vector Machine (Linear Kernel)	Number of PCs: 80 *C*: 100.0	0.960 ± 0.026
Support Vector Machine (RBF Kernel)	Number of PCs: 20 *C*: 100.0 *γ*: 0.001	0.972 ± 0.018
Logistic Regression	Number of PCs: 40 *C*: 10.0	0.961 ± 0.028
Decision Tree	Maximum depth: 40	0.969 ± 0.014
Random Forest	Number of estimators: 64	0.996 ± 0.006
Multilayer Perceptron	*α*: 1.0 Hidden layer size: (128,128,64)	0.962 ± 0.032

### Odor identification of spices through the free-hand measurement

**Figure 7:**
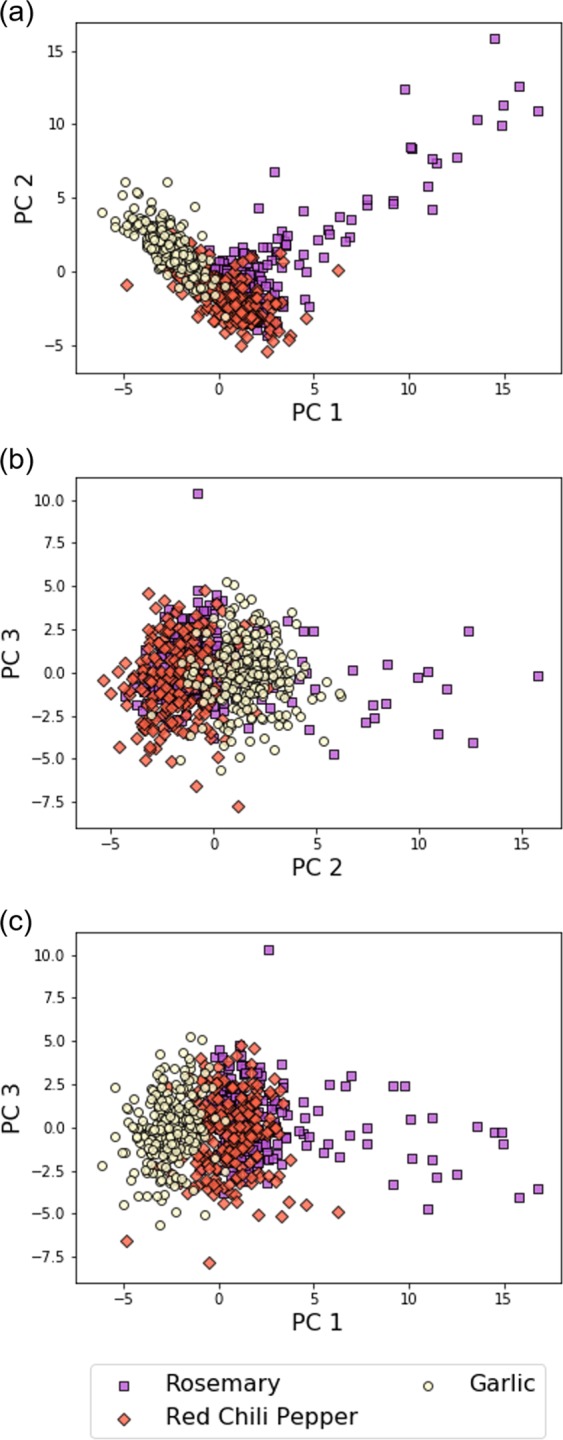
The scatter plots of PCA on the dataset of the odors of the spices and herbs obtained with MSS Chip II.

Not only vapors of solvents (single component gases) but also odors (multicomponent gas mixtures) are within the scope of this new gas identification protocol. To demonstrate odor identification through the free-hand measurement, we chose three spices as samples: rosemary, red chili pepper, and garlic. The odors of the spices were measured with MSS Chip II through the same experimental process as in the case of solvent vapors. The dataset of *K*_*m*,*n*_(*f*) was created from the measurement data and analyzed by PCA. Figure 7a–c show the scatter plots of PCA. The formation of clusters on the plots indicates that odors can be discriminated by *K*_*m*,*n*_(*f*). The results of the developed machine learning models are summarized in Table 4. As with the case of solvent vapors, the random forest-based model exceeds other models in accuracy (0.89 ± 0.04). These results demonstrate the odor identification with the compact measurement system consisting of only an MSS chip and electrical readout devices without any gas flow control.

**Table 4:** Results of classification models for spices using MSS Chip II.

Classifier	Optimized Parameters	Accuracy
Support Vector Machine (Linear Kernel)	Number of PCs: 40 *C*: 0.1	0.82 ± 0.05
Support Vector Machine (RBF Kernel)	Number of PCs: 80 *C*: 100.0 *γ*: 0.001	0.84 ± 0.03
Logistic Regression	Number of PCs: 80 *C*: 1.0	0.84 ± 0.05
Decision Tree	Maximum depth: 80	0.79 ± 0.07
Random Forest	Number of estimators: 1024	0.89 ± 0.04
Multilayer Perceptron	*α*: 10.0 Hidden layer size: (128,128,64)	0.84 ± 0.06

### Gas identification with different gas input patterns

**Figure 8:**
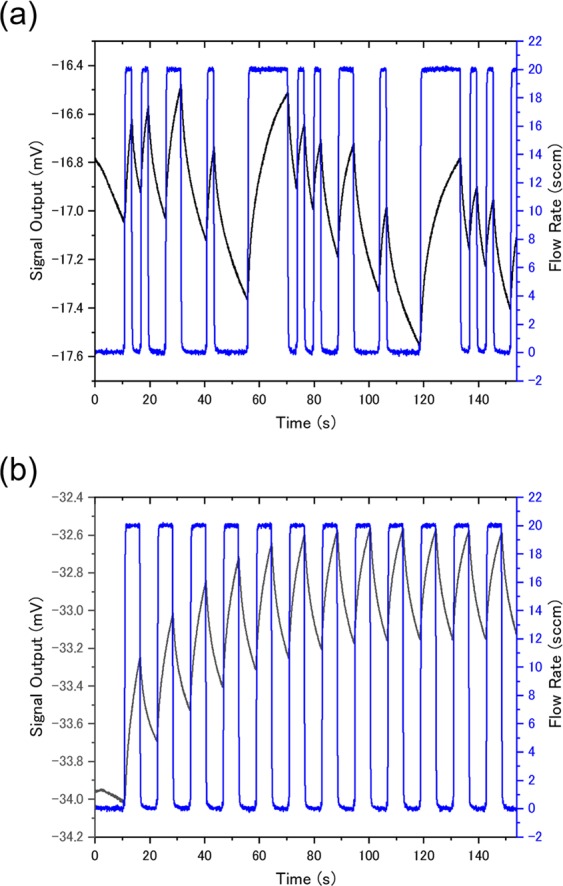
Sensing signals from channel 1 of MSS Chip II and the monitored flow rates of a sample gas (ethanol) for (**a**) the m-sequence pseudorandom sequence and (**b**) the rectangular sequence.

**Figure 9:**
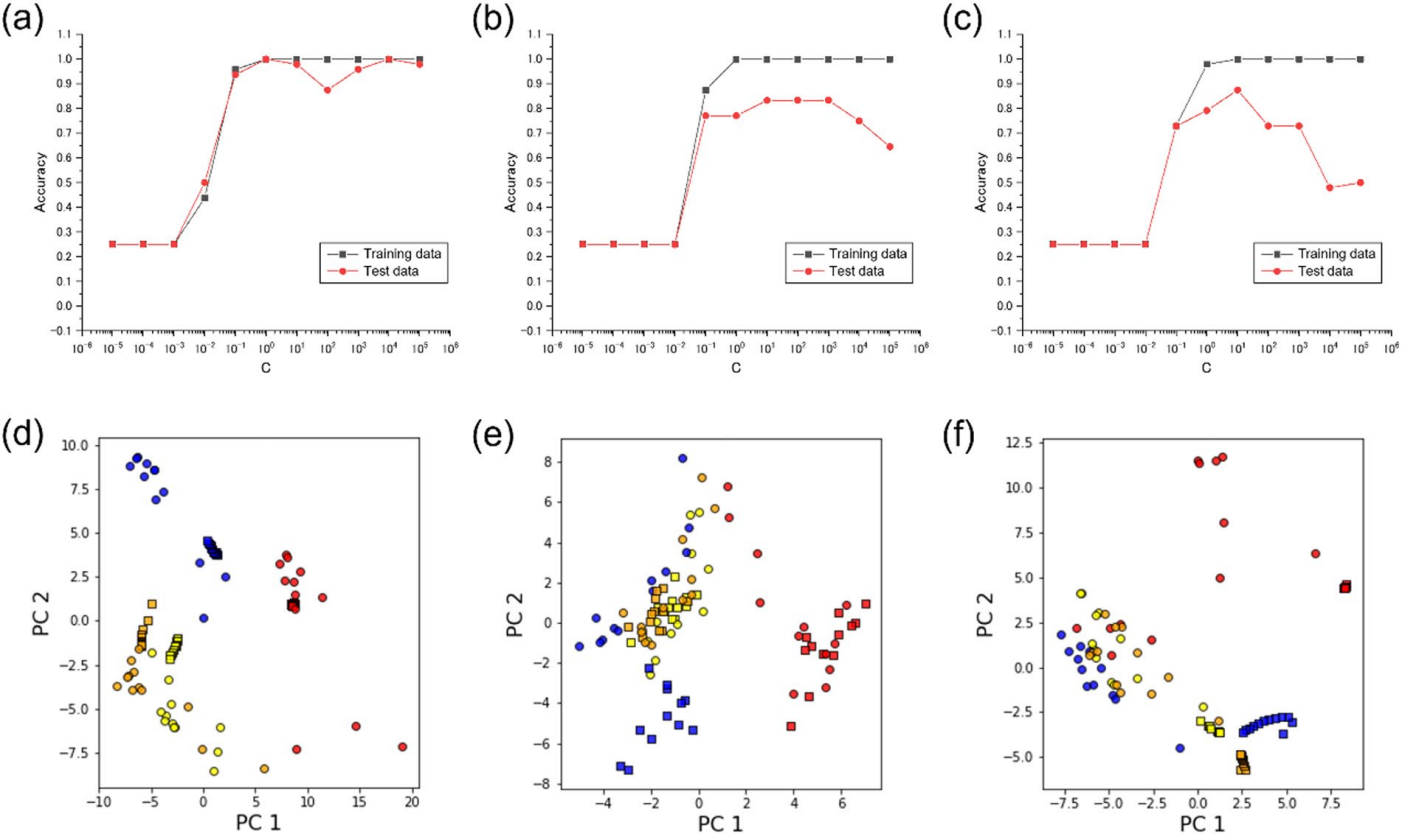
Accuracies for training and test datasets plotted against regularization parameter *C*; the datasets were generated through the analysis methods based on (**a**) TFR, (**b**) AR model, and (**c**) FFT. Scatter plots of PCA from the datasets based on (**d**) TFR, (**e**) AR model, and (**f**) FFT. Yellow, red, blue, and orange correspond to ethanol, water, heptane, and ethyl acetate, respectively. Circle and square markers represent the m-sequence pseudorandom sequence and the rectangular sequence, respectively.

Finally, we conducted gas sensing measurements with different types of gas flow sequences and developed classification models from the measurement data in order to explicitly show that TFR is adaptable to any gas input pattern. Headspace gases of the four solvent vapors were injected to MSS Chip II with a gas flow line equipped with MFCs to generate definitely different gas input patterns. Two different gas flow sequences were employed for the gas measurements: the m-sequence pseudorandom sequence (Fig. 8a) and the rectangular sequence (Fig. 8b). The sensing data obtained from the m-sequence pseudorandom sequence and the rectangular sequence were used for training and testing classification models, respectively. Note that the monitored gas flow rates were not used for analysis. Logistic regression was employed as a classifier. Figure 9a shows the results. The classification accuracy for training and test data are plotted against the regularization parameter *C*. For *C* > 10^−1^, classification models which show high accuracy for both training and test data are developed. Therefore, it is demonstrated that gas species can be identified independently of the gas input pattern.

To compare the TFR-based analysis with other typical analytical methods, we also developed two models based on AR models and fast Fourier transform (FFT). The results are shown in Fig. 9b,c. The suppressed accuracies in the test data for these analysis methods are attributed to the intrinsic difference from the TFR-based analysis, which is independent from the gas input pattern. Since the conventional analytical methods including AR models and FFT depend on gas input patterns, classification models which can be adapted to the different gas input patterns could not be developed. Accordingly, these approaches inevitably result in low accuracies for non-trained gas input patterns, which are common for practical measurements without gas flow control. The results of PCA on each dataset also indicate the robustness of TFR to gas input patterns. Figure 9d–f show the PCA scatter plots of the TFR-, AR-, and FFT-datasets, respectively. The colors of the markers represent the solvents; yellow, red, blue, and orange correspond to ethanol, water, heptane, and ethyl acetate, respectively. The circle and square markers are the data obtained from the m-sequence pseudorandom sequence and the rectangular sequence, respectively. While there is a slight overlap between ethanol and ethyl acetate, TFR-dataset exhibits the highest separation even though the data obtained with the two different gas input patterns are mixed. The details of the measurements and the analysis procedure are described in Supplementary Information.

## Conclusion

In this study, we have proposed a new gas identification protocol that does not require any gas flow controls. The key figure of this protocol is the data analysis method based on the TFR, which is independent of the gas input pattern and intrinsic to the combination of a sensor and a gas species. As TFR can be estimated only from the sensing signals of a gas sensor array (or a multichannel gas sensor chip), gas species can be identified without gas flow control units including pumps or MFCs. Combined with a miniaturized gas sensor chip, this gas identification protocol realizes the free-hand measurement, in which samples are measured simply by holding the sensor chip near the sample. To demonstrate the gas identification through the free-hand measurement, we developed a compact measurement system based on MSS and developed machine learning models for gas identification. By using the MSS chip coated with functionalized nano-/micro particles, we demonstrated the identification of not only solvent vapors but also odors of spices through the free-hand measurement with high accuracies. As MSS can utilize diverse materials for their receptor layers, the combination of the receptor materials in an MSS chip can be further optimized on the basis of the chemical composition of target odors. The robustness of the TFR-based analysis to gas input patterns was confirmed by building and validating classification models from measurement data obtained with explicitly different gas input patterns.

This gas identification protocol is not limited to MSS but applicable to any miniaturized gas sensor array or multichannel gas sensor chip. As gas flow control units such as pumps are not required in this protocol, a compact artificial olfactory system consisting of only a sensor chip and an electrical readout system can be realized, leading to the implementation of artificial olfaction in portable electronics and even in wearable devices. We believe this study will contribute to the realization of practical artificial olfaction.

## Methods

### Fabrication of MSS Chip I

The polymers were dissolved in solvents; poly(vinylidene fluoride), polysulfone, and polycaprolactone were dissolved in N,N-dimethylformamide (DMF), while poly(4-methylstyrene) was dissolved in trichloroethylene. The concentration of the solutions was set at 1.0 g/L. The solutions are delivered onto the channels of MSS by an inkjet spotter (LaboJet-500SP, MICROJET Corporation). The details of the inkjet spotting are described in Supplementary Information.

### Signal read-out system

The sensing signals of MSS are obtained as resistance changes in the piezoresistors embedded in the four beams. The four piezoresistors form a Wheatstone bridge. To acquire the sensing signals from the MSS chip, a bridge voltage of −0.5 V was applied to the Wheatstone bridge circuits in the MSS chip with a digital-to-analog converter module (NI-9269, National Instruments). The sensing signals were collected with an analog-to-digital converter module (NI-9214, National Instruments). The sampling rate was set at 20 Hz.

### Free-hand measurement

We conducted the free-hand measurements on each sample for 8 times. To improve the generalization performance of developed models, the same sample was not measured for two consecutive times. The MSS sensor chips were not cleaned (e.g. exposure to dried air, annealing) during the intervals of each measurement. All the experiments were done in a fume hood for safety, and the measurement data were acquired over two days.

### Development of machine learning models

From the obtained measurement data (i.e. 32 files of time-series data), we built machine learning models for gas identification based on TFR. The measurement data—the time-series data of 90 seconds—were divided by *t*_*m*_; hence, the total number of the divided data becomes 90/*t*_*m*_. From each segmented data, *K*_*m*,*n*_(*f*) was calculated according to Eq. (5). FFT was applied on each segmented data, which was zero-meaned and multiplied by the Hann function in advance. Then, *K*_*m*,*n*_(*f*) was calculated for all the six combinations: (*m*, *n*) = (1, 2), (1, 3), (1,4), (2, 3), (2, 4), (3, 4). As the sampling rate and the time length of each data were 20 Hz and *t*_*m*_ seconds, respectively, the components of *K*_*m*,*n*_(*f*) exist at 0, 1/*t*_*m*_, 2/*t*_*m*_, …, 10 Hz as complex numbers. Some frequency components were selected from each *K*_*m*,*n*_(*f*), and all the selected components were concatenated. In this study, *t*_*m*_ was set at 3.0 seconds, and the frequency components ranging from 0.333 to 3.333 Hz were used. The complex number was divided into the absolute value and the argument. Therefore, the sample size and the dimension of the dataset are 960 and 120, respectively. The optimization scheme for the feature selection is described in Supplementary Information. Based on the dataset of *K*_*m*,*n*_(*f*), we developed machine learning models for classifying the gas species.

## Supplementary information

Identification of Solvent Vapors Through Free-Hand Measurement

Supplementary Information
