# Supplementary material for: Free-hand gas identification based on transfer function ratios without gas flow control
Source: Sci Rep. 2019 Jul 5;9:9768. doi: 10.1038/s41598-019-46164-1 (PMC6611792; doi:10.1038/s41598-019-46164-1)
Supplement: Supplementary file 2 — Supplementary Information [file 41598_2019_46164_MOESM2_ESM.docx]

Free-hand gas identification based on transfer function ratios without gas flow control

Gaku Imamura^1,2^*, Kota Shiba^1,2^, Genki Yoshikawa^1,2,3^, and Takashi Washio^4^

*^1^World Premier International Research Center Initiative (WPI), International Center for Materials Nanoarchitectonics (MANA), National Institute for Materials Science (NIMS), Tsukuba, Ibaraki, 305-0044, Japan*

*^2^Center for Functional Sensor & Actuator (CFSN), National Institute for Materials Science (NIMS), Tsukuba, Ibaraki, 305-0044, Japan*

*^3^Materials Science and Engineering, Graduate School of Pure and Applied Science, University of Tsukuba, Tennodai 1-1-1 Tsukuba, Ibaraki 305-8571, Japan*

*^4^The Institute of Scientific and Industrial Research, Osaka University, Mihogaoka 8-1, Ibaraki, Osaka 567-0047. Japan*

*E-mail: IMAMURA.Gaku@nims.go.jp

**Supplementary Information**

# Coating Method

Receptor layers were coated by an inkjet spotter (LaboJet-500SP, MICROJET Corporation). The parameters for inkjet spotting were optimized as summarized in Table S1.

Table S1. Parameters for inkjet spotting for coating receptor layer.

| Parameter | Value |
| --- | --- |
| Injection speed | ~5 m/s |
| Volume of a droplet | ~300 pL |
| Stage temperature | 80 °C |
| Number of shots | 300 shots |

The topography of the receptor materials was investigated with a profilometer (Dektak, Bruker Corporation). The receptor materials are coated on silicon substrates with the same inkjet spotting procedure. The results are depicted in Figs. S1 and S2. The thickness of the receptor materials is in the range of 1 to 50 μm.


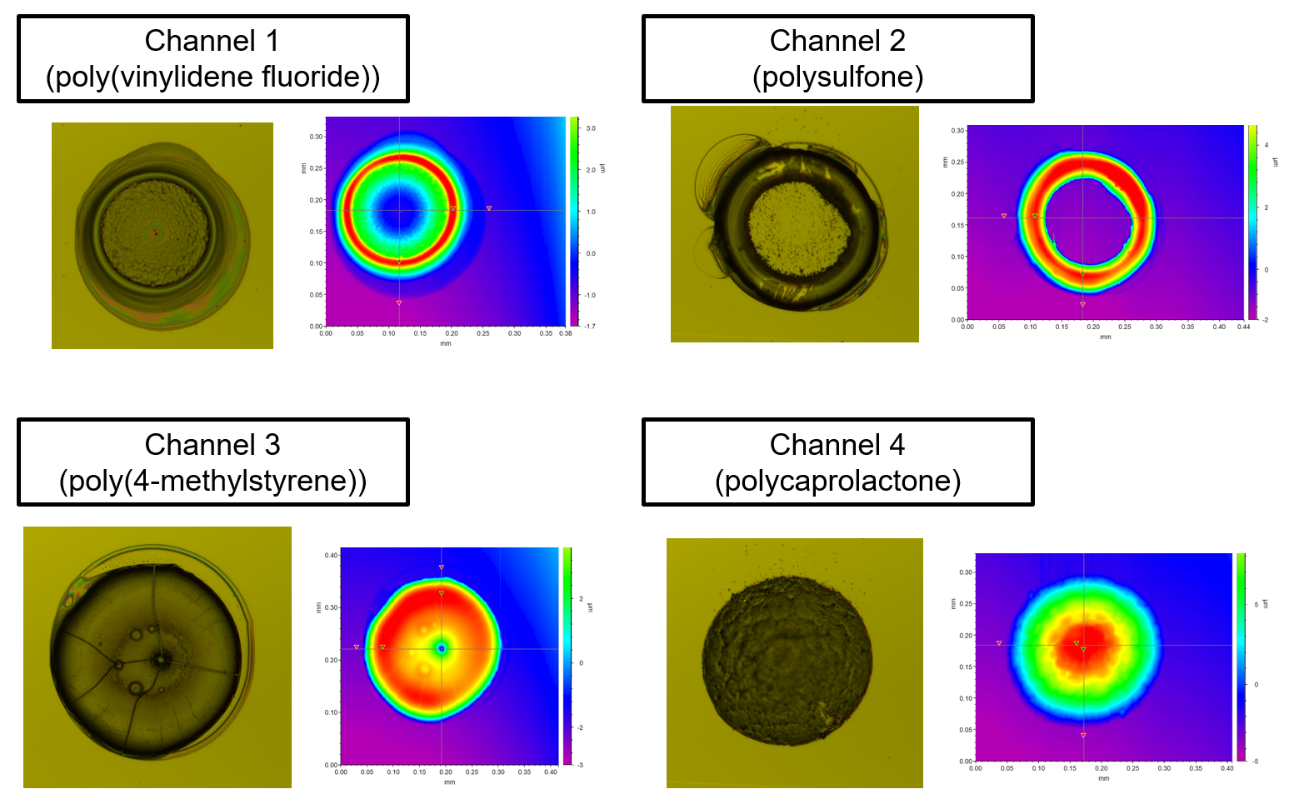


**Figure S1** Topography of the receptor materials coated on MSS Chip I.


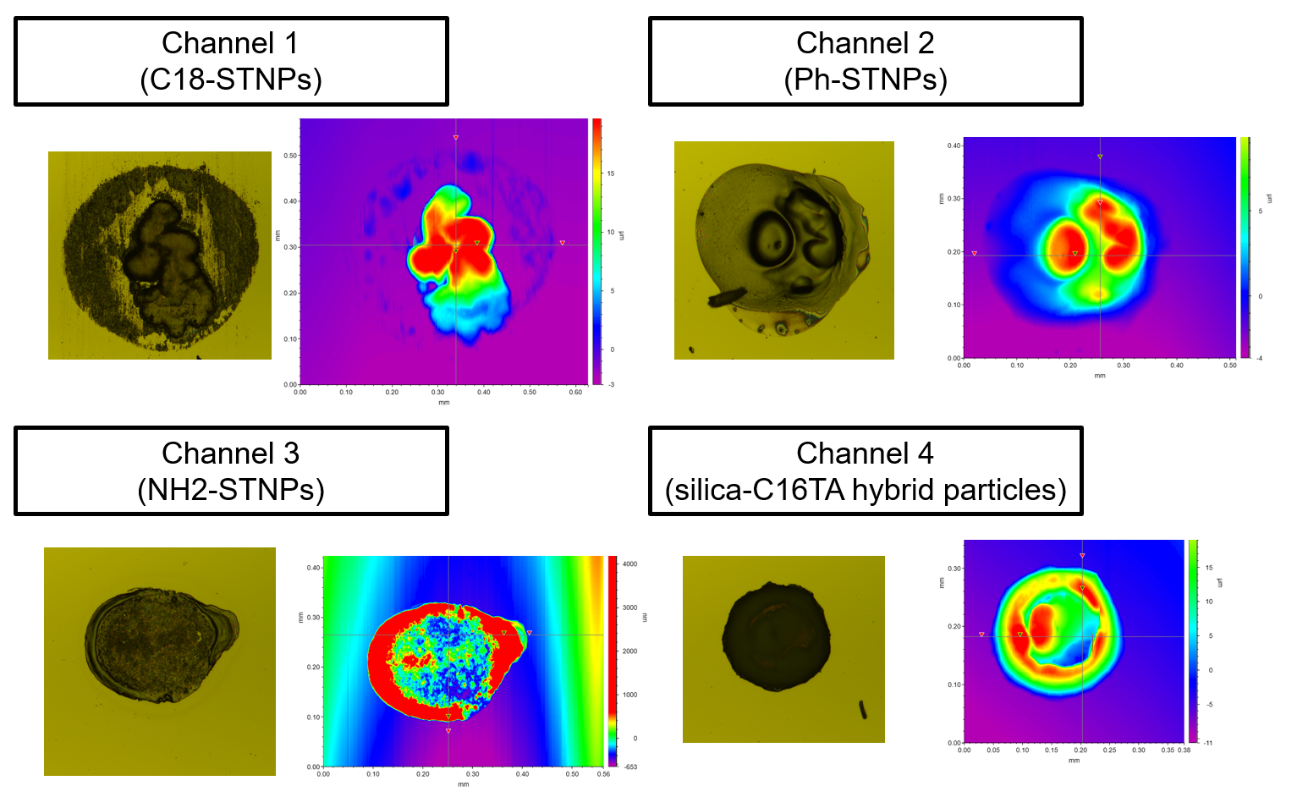


**Figure S2** Topography of the receptor materials coated on MSS Chip II.

# Free-Hand Measurement

In the free-hand measurement, sensing signals were manually obtained by moving an MSS chip near a sample (see the main manuscript). The chips were randomly moved so that the sensing signals contain wide range of frequency components.

## Sensing Responses of MSS Chip I to the Solvent Vapors Through the Free-Hand Measurement


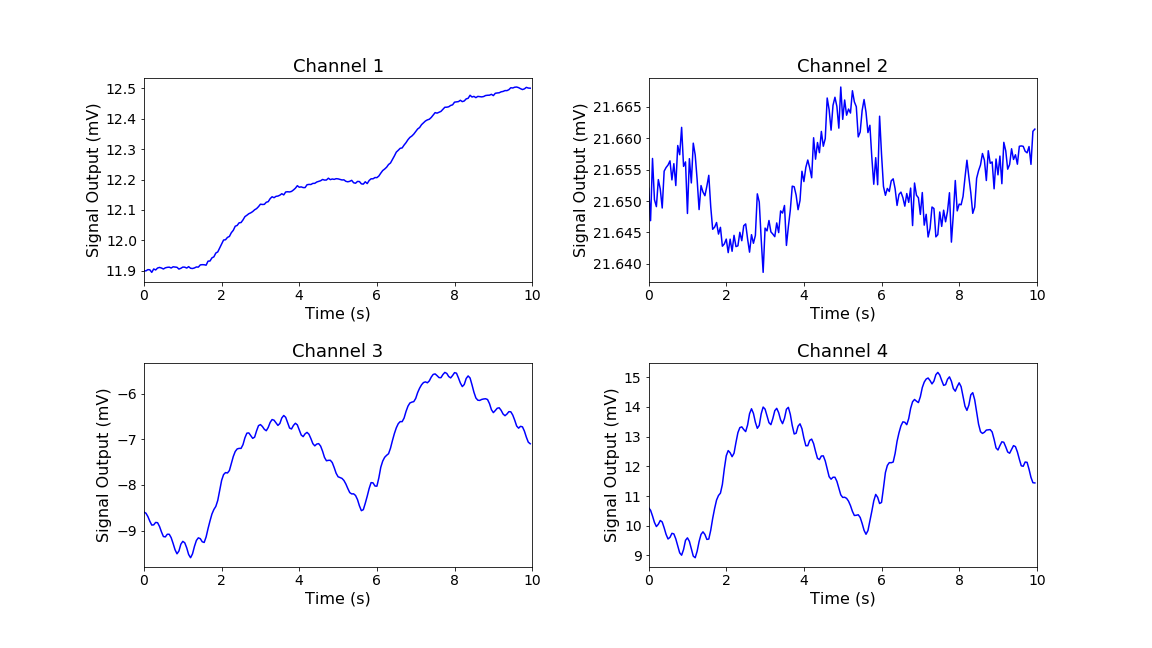


**Figure S4** Sensing responses of MSS Chip I to ethanol vapor with the free-hand measurement.


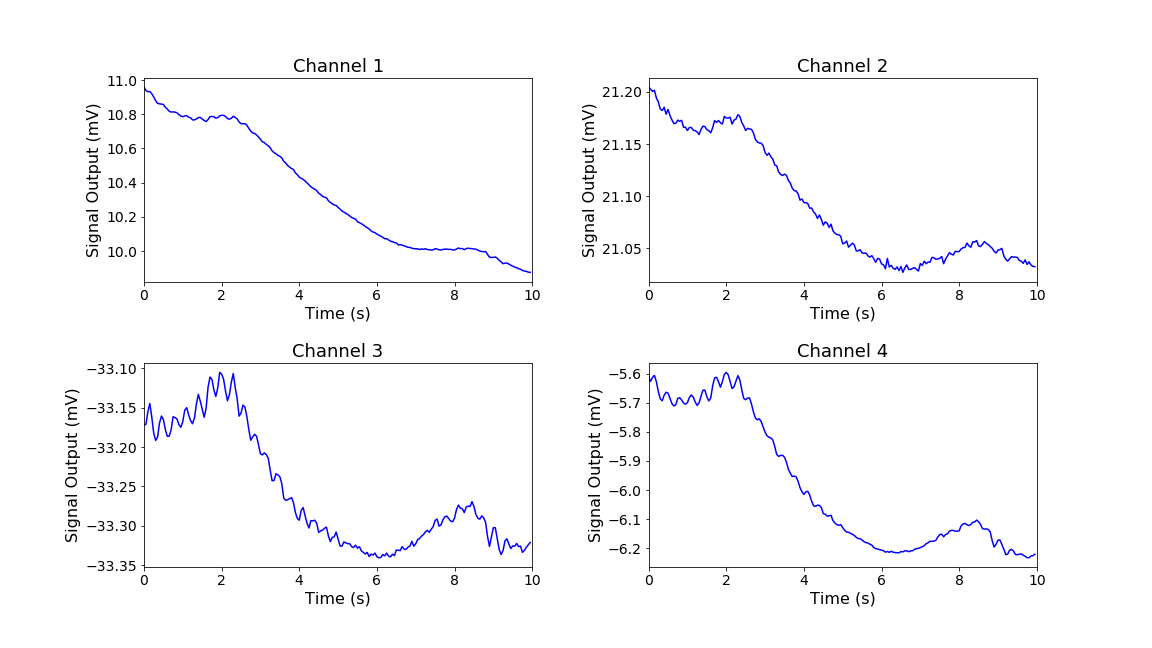


**Figure S5** Sensing responses of MSS chip I to water vapor with the free-hand measurement.


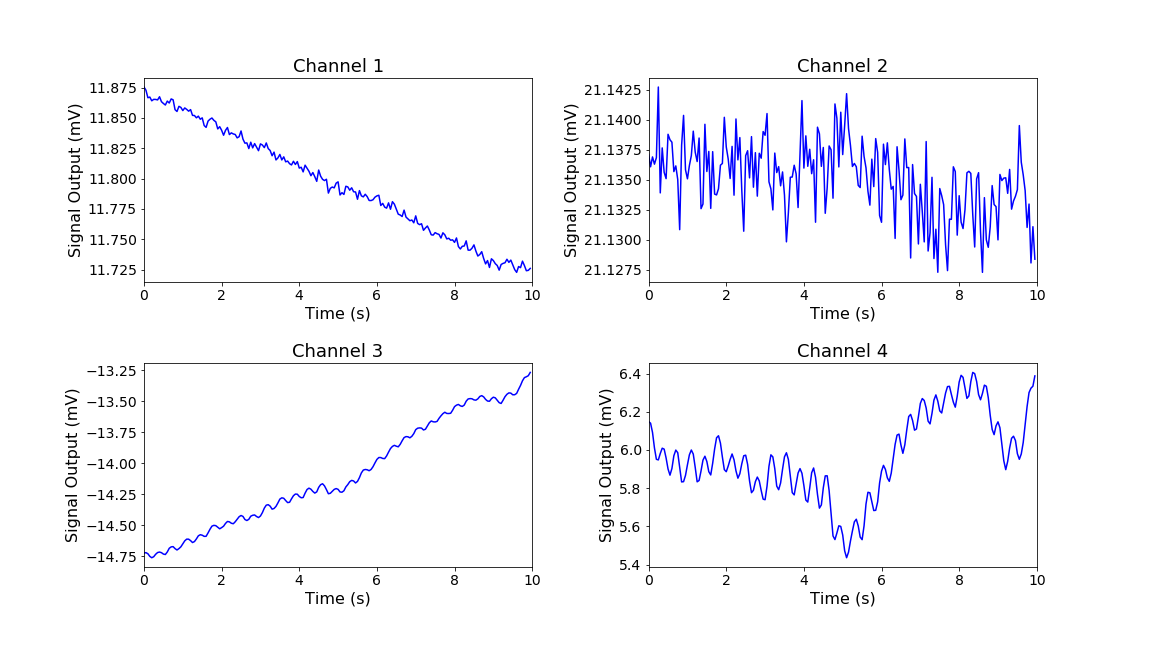


**Figure S6** Sensing responses of MSS chip I to heptane vapor with free-hand measurement.


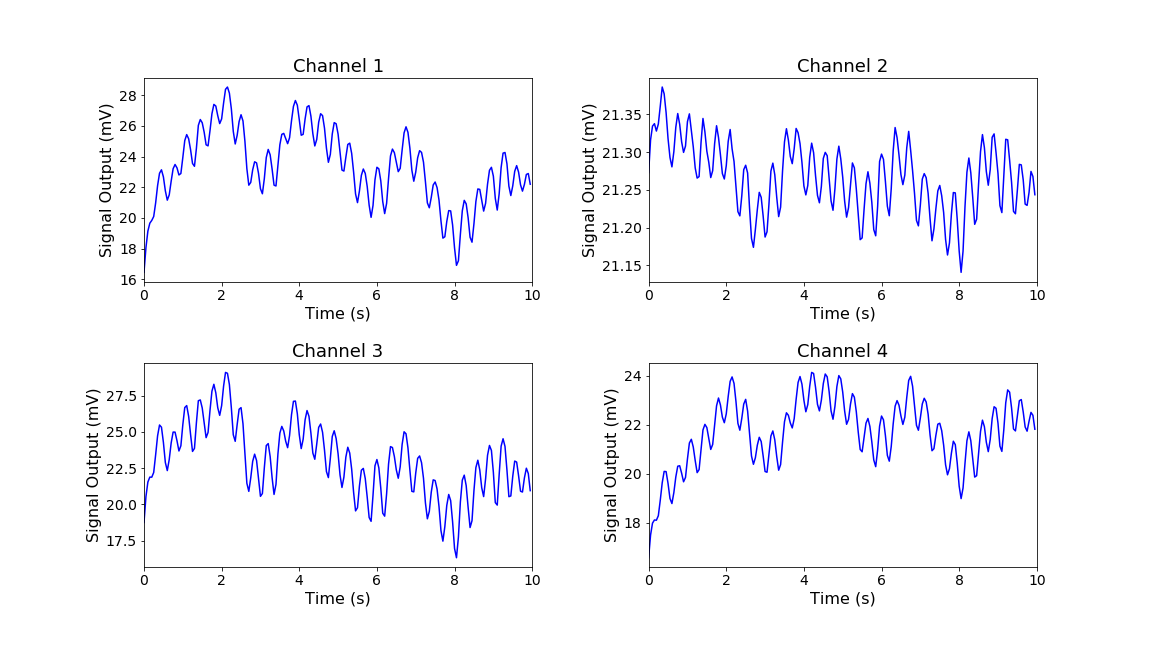


**Figure S7** Sensing responses of MSS chip I to ethyl acetate vapor with the free-hand measurement.

## Sensing Responses of MSS Chip II to the Solvent Vapors Through the Free-Hand Measurement


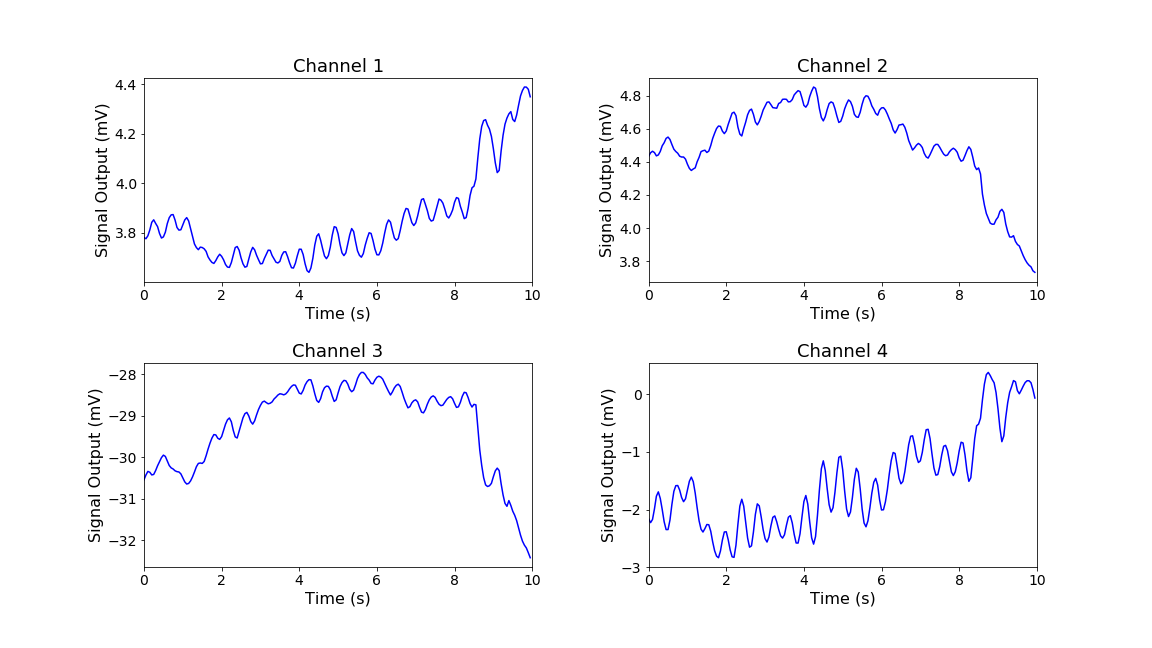


**Figure S8** Sensing responses of MSS chip II to ethanol vapor with the free-hand measurement.


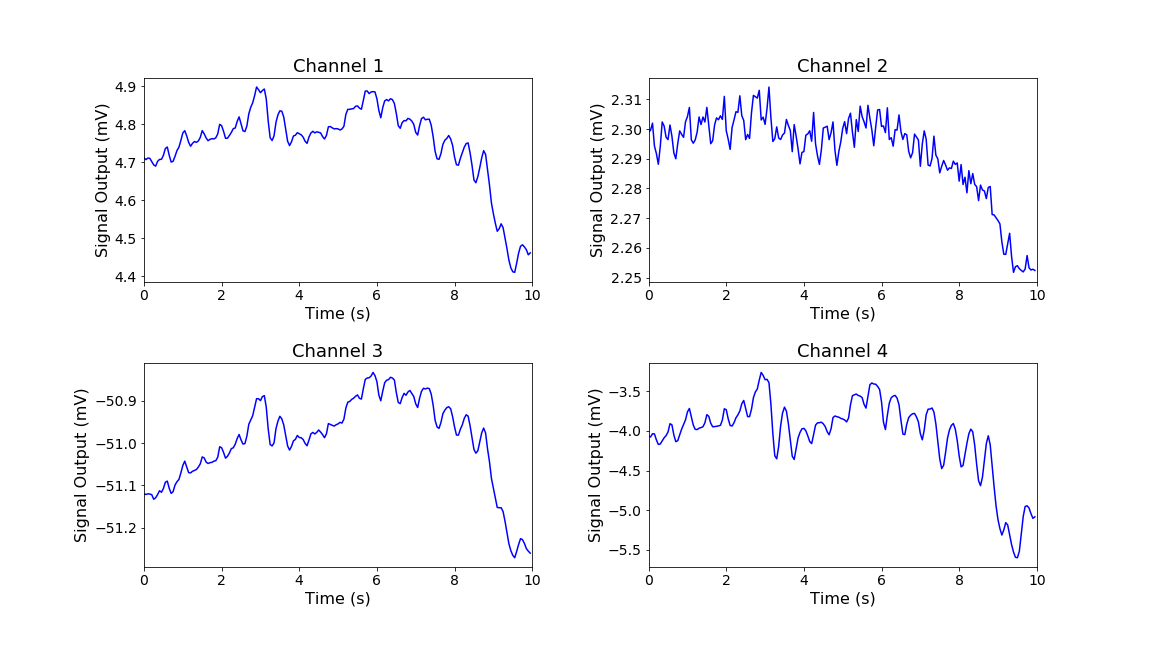


**Figure S9** Sensing responses of MSS chip II to water vapor with the free-hand measurement.


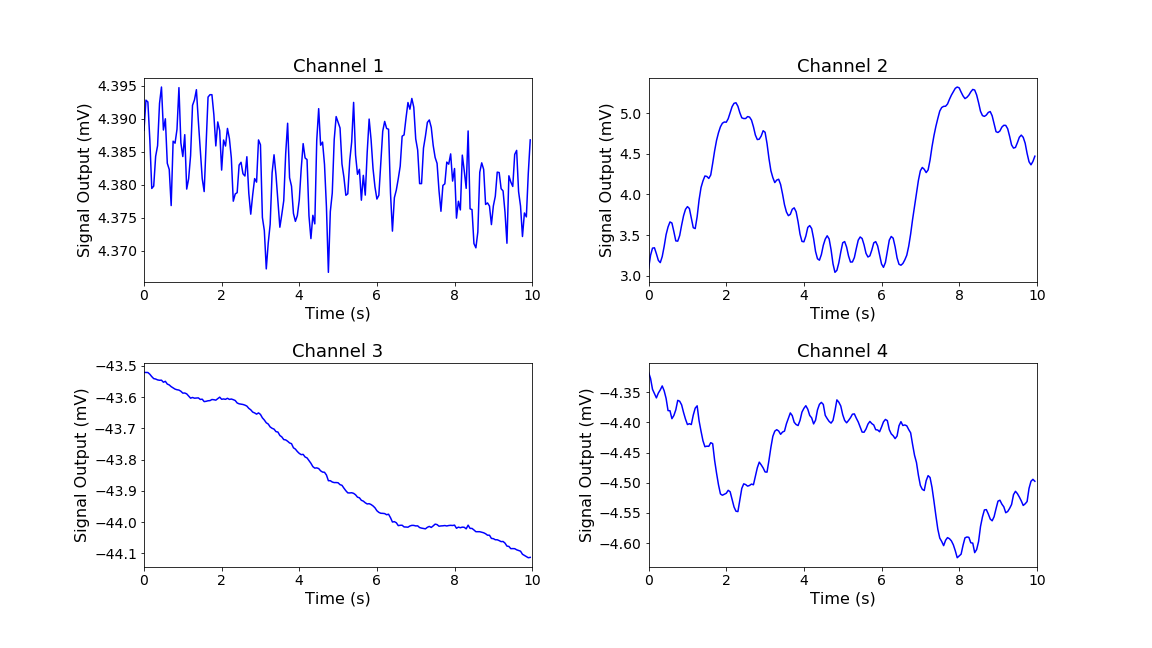


**Figure S10** Sensing responses of MSS chip II to heptane vapor with the free-hand measurement.


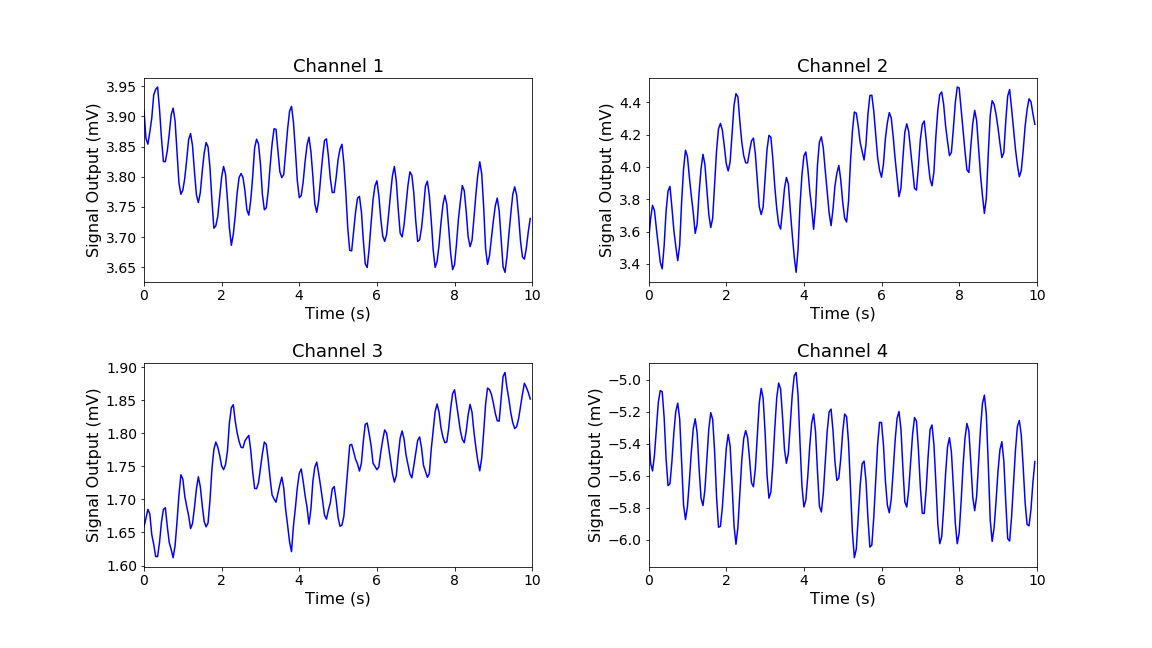


**Figure S11** Sensing responses of MSS chip II to ethyl acetate vapor with the free-hand measurement.

# Plots of $K_{m,n}\left( f \right)$

(a)

(b)


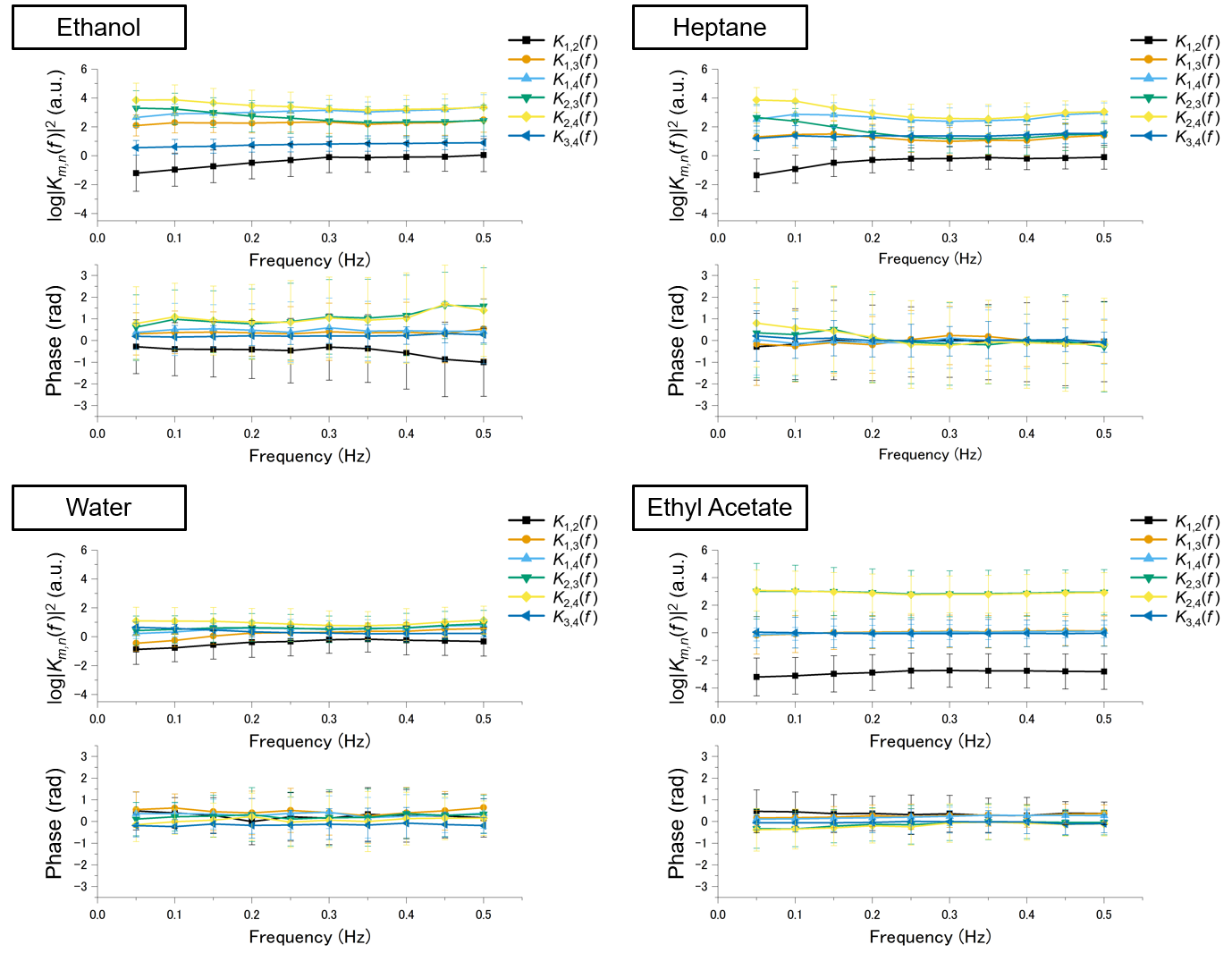

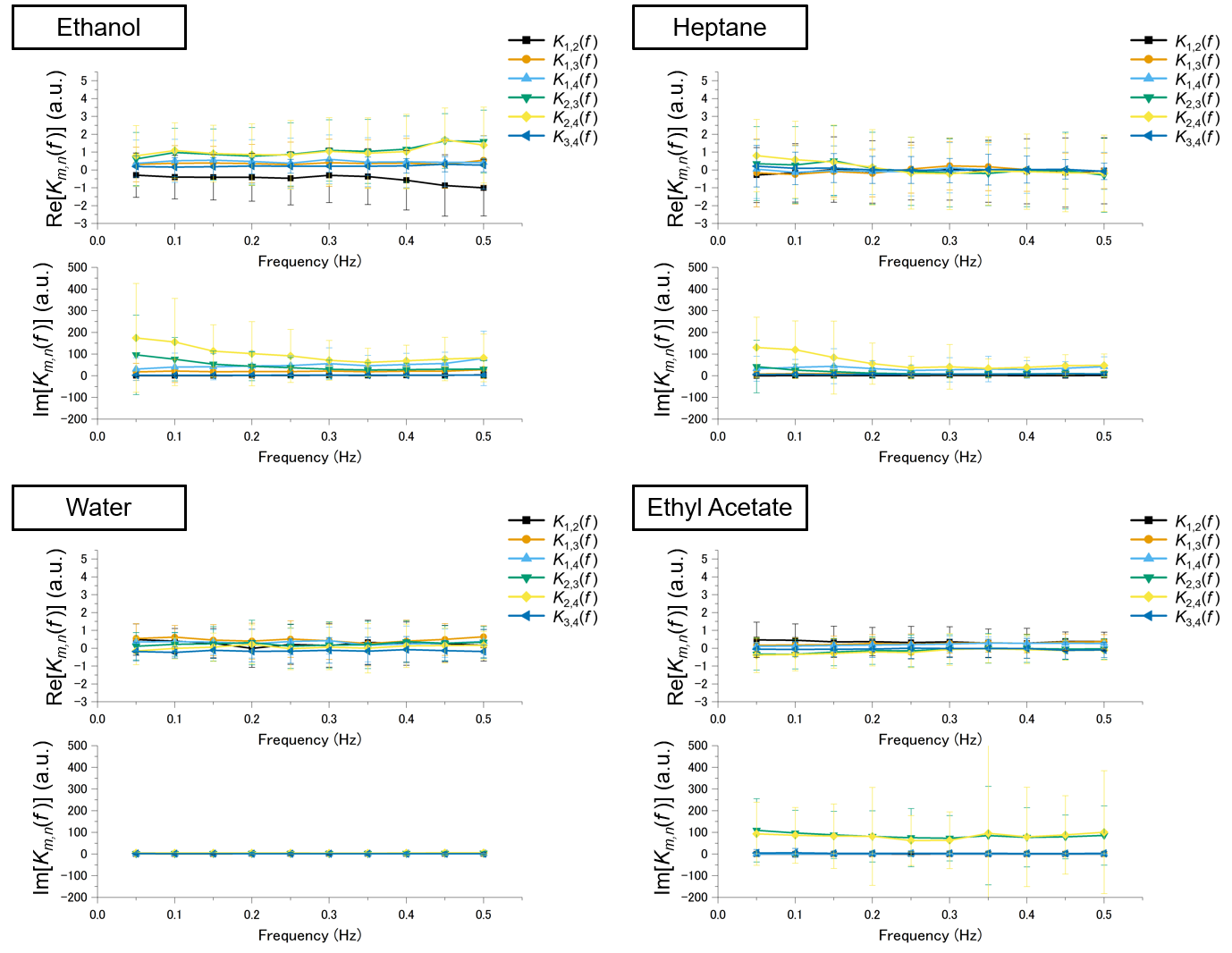


**Figure S12** Plots of $K_{m,n}\left( f \right)$ of MSS Chip I for each solvent vapor in (a) Cartesian and (b) polar coordinate form.

(a)

(b)


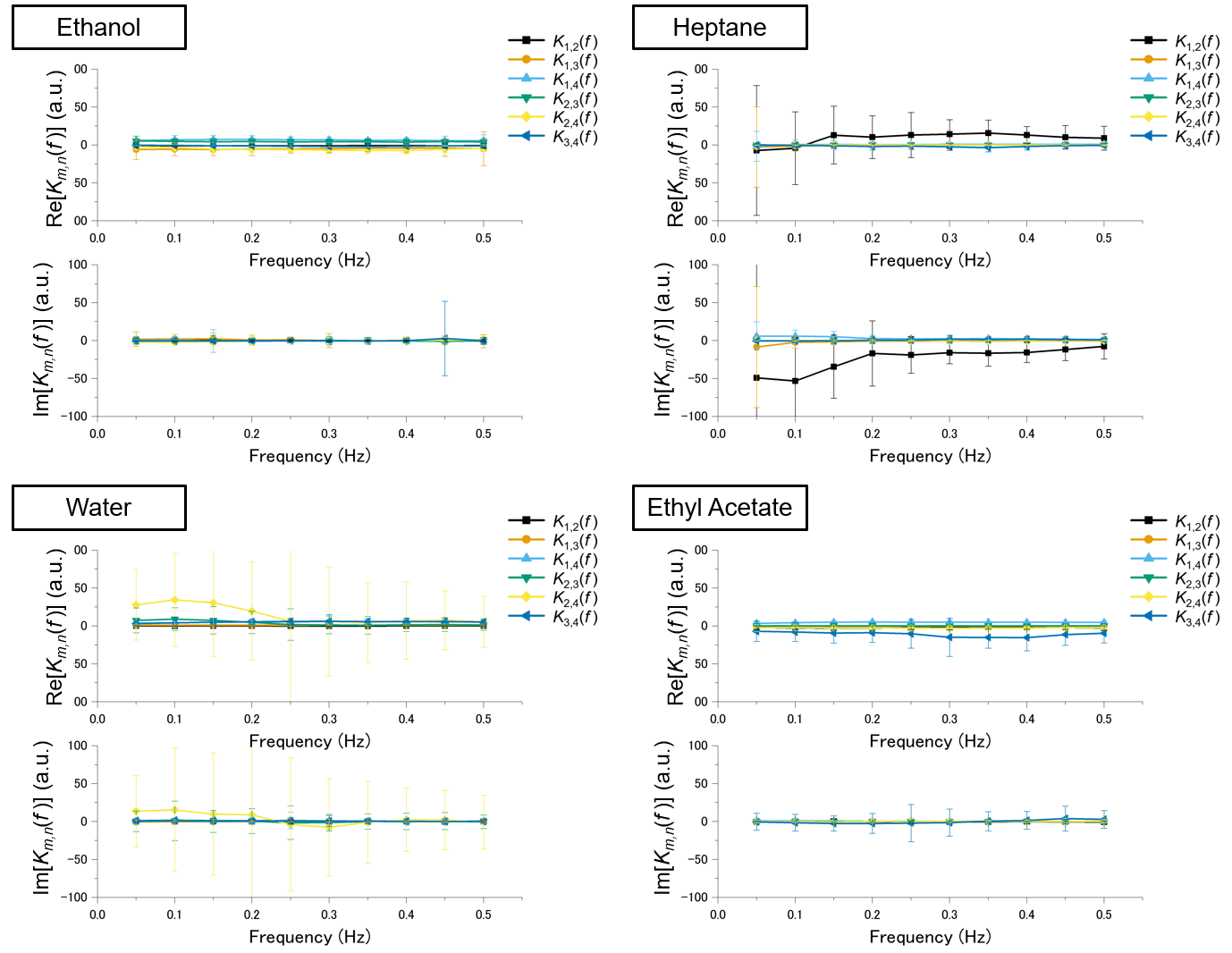

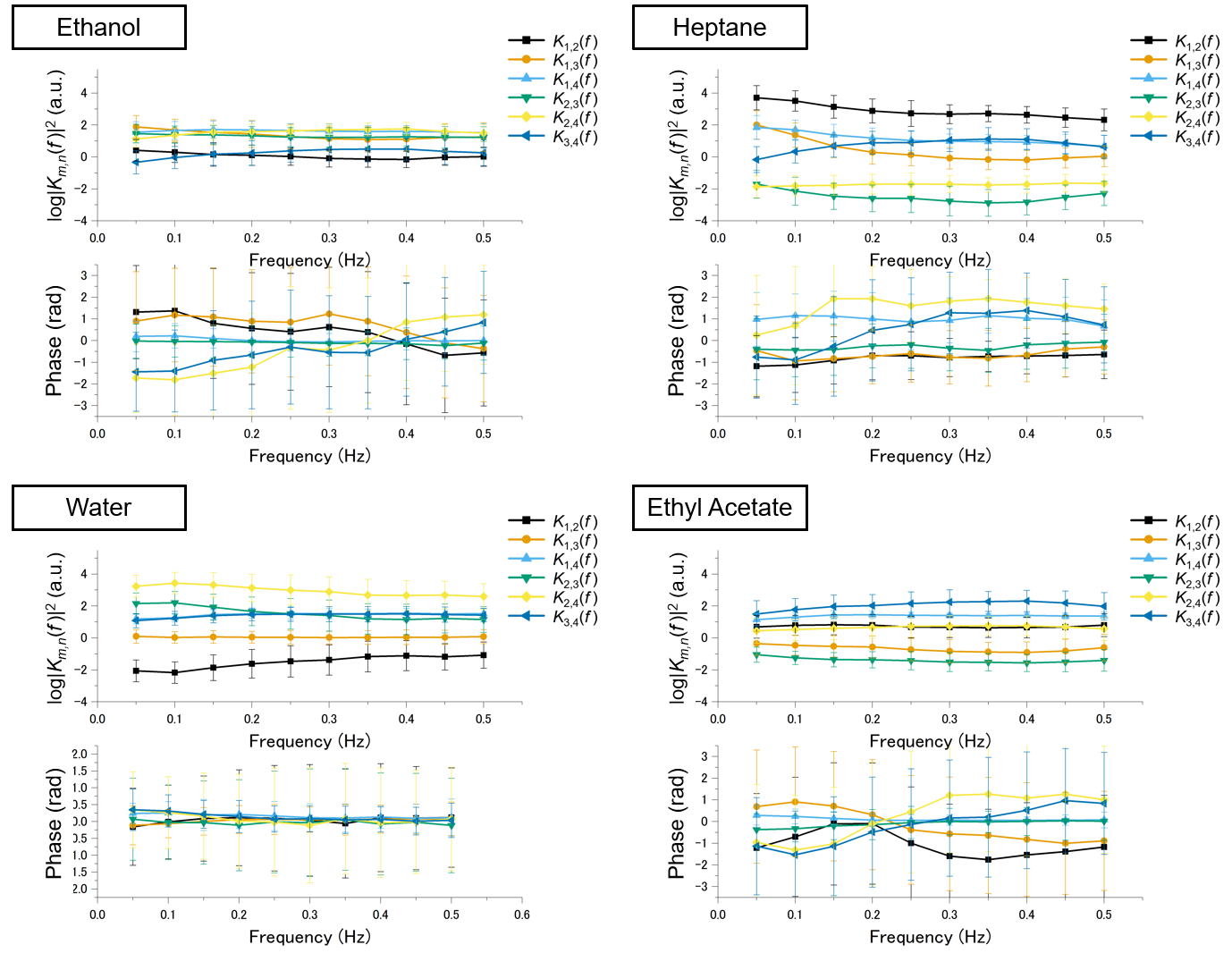


**Figure S13** Plots of $K_{m,n}\left( f \right)$ of MSS Chip II for each solvent vapor in (a) Cartesian and (b) polar coordinate form.

# Optimization of Feature Selection

To develop valid and reliable machine learning models, feature selection plays an important role as well as feature extraction. From the frequency components of $K_{m,n}\left( f \right)$, we made seven datasets of selected features and built machine learning models. In this optimization process, we evaluated the accuracy through 10×10 CV in which training and test datasets were randomly split. Note that model building and evaluation procedure is different from the one performed in the main script. Table S2 summarizes the datasets. The dataset obtained from the MSS Chip II is also described as dataset No. 8 in Table S2 for comparison. We modified the measurement time $t_{m}$, selected frequency components, and the form of complex number. Figure S14a shows the result of the accuracies of the machine learning models built from the datasets. Accuracies did not differ noticeably between the datasets except dataset No.5, which results in the lowest accuracies of the seven datasets. Since only the high frequency components are selected for dataset No.5, the dataset may contain noise components rather than the response originated from the gas sorption/desorption dynamics.

Table S2. Details of the datasets

| Dataset | Measurement time, $\boldsymbol{t}_{\boldsymbol{m}}\boldsymbol{(s)}$ | Frequency Components  (Hz) | Form of complex numbers | Sample size | Number of dimensions |
| --- | --- | --- | --- | --- | --- |
| 1 | 3 | (0.333, 0.666, …, 3.333) | Polar form | 960 | 120 |
| 2 | 3 | (0.333, 0.666, …, 6.667) | Polar form | 960 | 240 |
| 3 | 5 | (0.200, 0.400, …, 2.000) | Polar form | 576 | 120 |
| 4 | 3 | (0.333, 1.333, …, 9.333) | Polar form | 960 | 120 |
| 5 | 3 | (6.667, 7.000, …, 9.667) | Polar form | 960 | 120 |
| 6 | 3 | (0.333, 0.666, …, 3.333) | Cartesian form | 960 | 120 |
| 7 | 3 | (0.333, 0.666, …, 3.333) | Absolute values | 960 | 60 |
| (8) | 3 | (0.333, 0.666, …, 3.333) | Polar form | 720 | 120 |


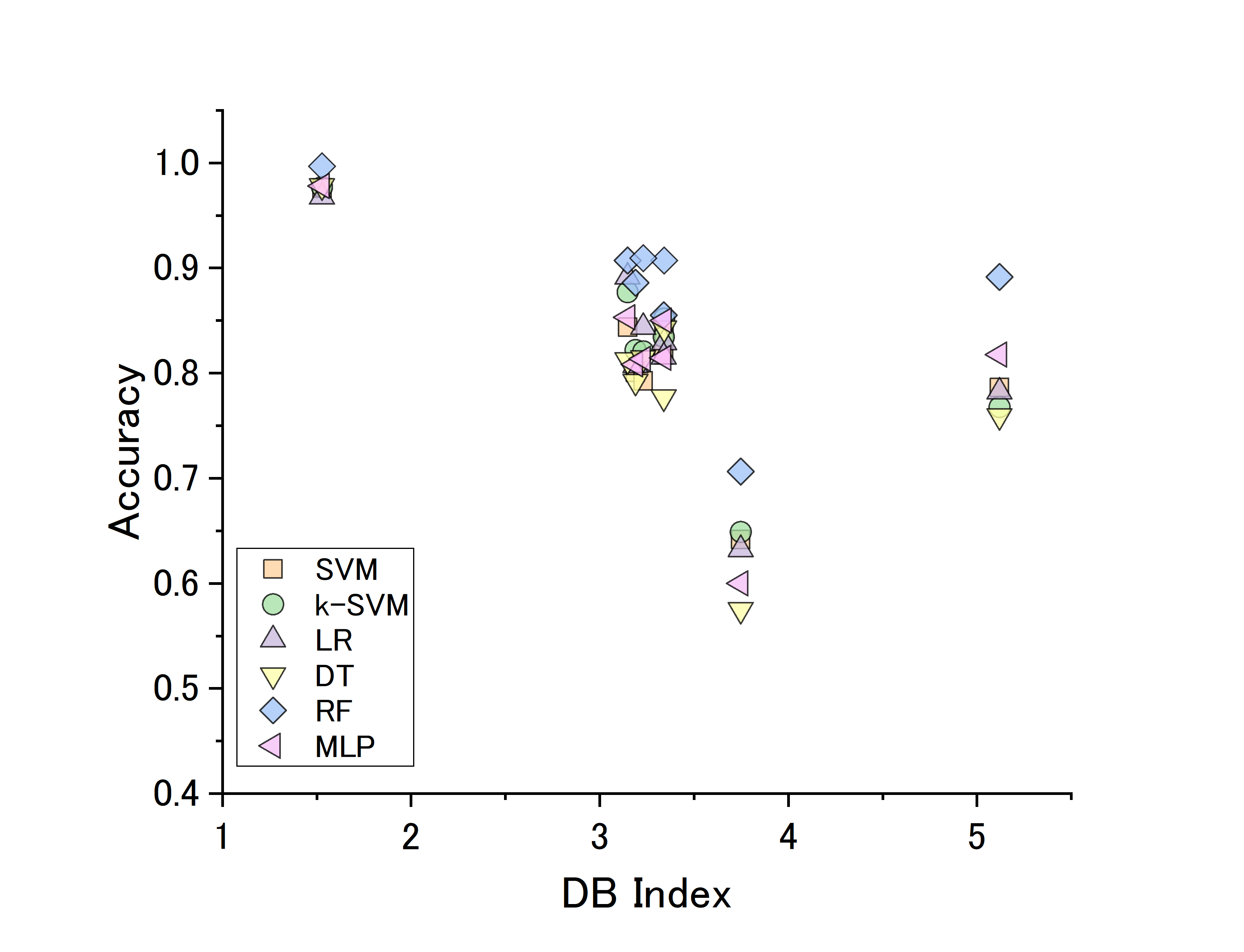

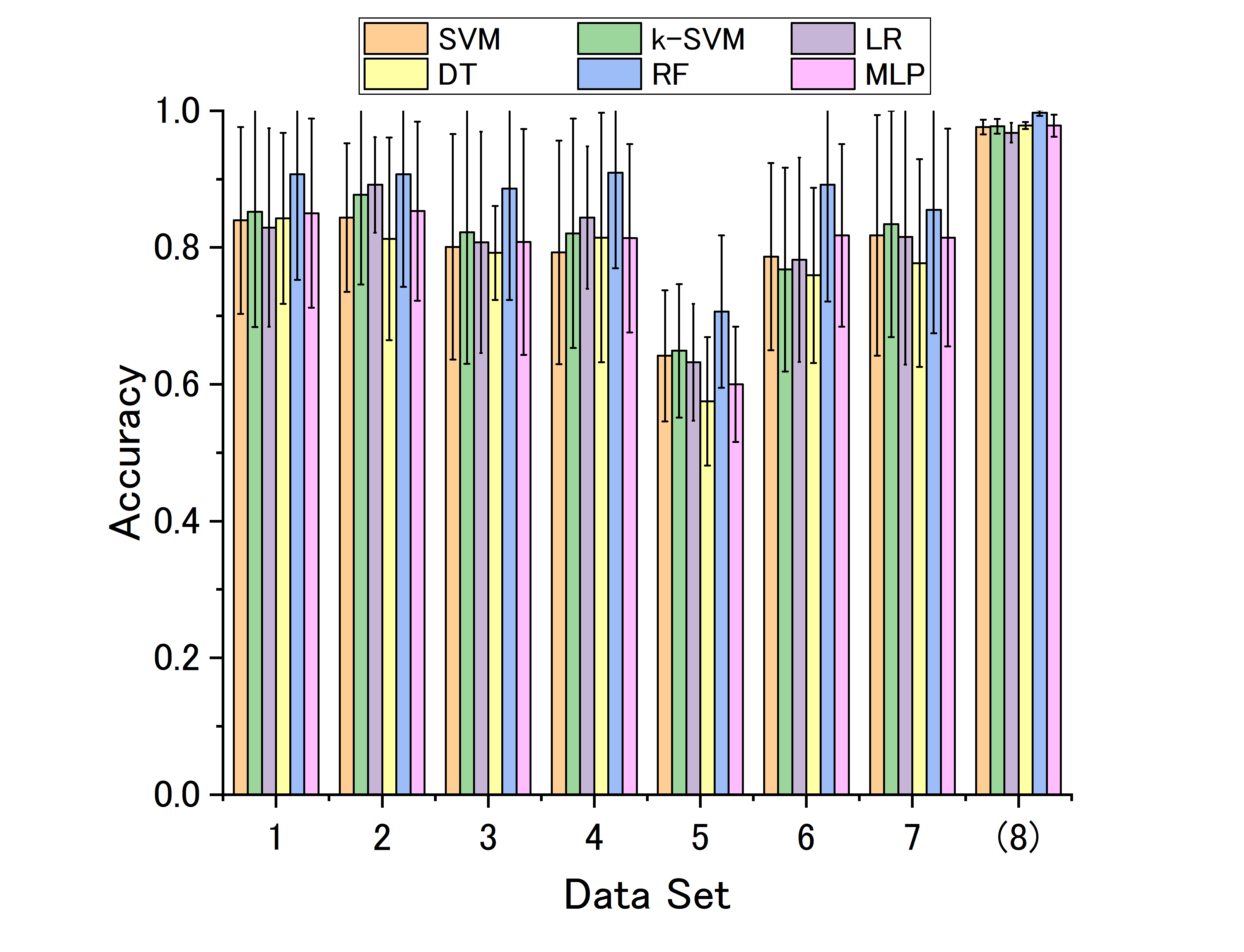


(b)

(a)

**Figure S14** (a) Classification accuracies of the models developed from the datasets. (b) The plot of classification accuracy against the DB index.

We also estimated the cluster quality of the datasets by the Davies–Bouldin (DB) index (see the main manuscript for the definition of the DB index). The accuracies of the machine learning models are plotted against the DB indices of the datasets in Fig. S14b. The DB index does not vary significantly between the datasets except dataset No. 6. For any classifier, there is no clear relationship between the DB index and the identification accuracy. As reported in the previous studies, the cluster quality does not assure a high classification accuracy in developing machine learning models ^S1,S2^.

Details of the developed machine learning models and the DB indices of the datasets are summarized in Table S3.

1. M. Pardo and G. Sberveglieri, Sensors and Actuators B: Chemical 123 (1), 437-443 (2007).
2. T. Nowotny, A. Z. Berna, R. Binions and S. Trowell, Sensors and Actuators B: Chemical 187, 471-480 (2013).

| Multilayer Perceptron | Random Forest | Decision Tree | Logistic Regression | Support Vector Machine  (RBF Kernel) | Support Vector Machine  (linear kernel) | DB index | Classifier |  |
| --- | --- | --- | --- | --- | --- | --- | --- | --- |
| 0.85±0.14  *α*: 1.0  Hidden layer size: (128, 128, 64) | 0.91±0.15  Number of estimators: 512 | 0.84±0.13  Maximum depth: 5 | 0.83±0.15  *N*_PC_: 20  *C*: 1.0 | 0.85±0.17  Table S3. Summary of machine learning models for the different feature sets.  *N*_PC_: 20  *C*: 100.0  *γ*: 0.001 | 0.84±0.14  *N*_PC_: 20  *C*: 1.0 | 3.34 | Accuracy  Optimized Parameters | 1 |
| 0.85±0.13  *α*: 10.0  Hidden layer size: (128, 128, 64) | 0.91±0.16  Number of estimators: 512 | 0.81±0.15  Maximum depth: 50 | 0.89±0.07  *N*_PC_: 20  *C*: 10.0 | 0.88±0.13  *N*_PC_: 40  *C*: 10.0  *γ*: 0.001 | 0.84±0.11  *N*_PC_: 20  *C*: 1.0 | 3.15 |  | 2 |
| 0.81±0.17  *α*: 0.1  Hidden layer size: (128, 128, 64) | 0.89±0.16  Number of estimators: 64 | 0.79±0.07  Maximum depth: 20 | 0.81±0.16  *N*_PC_: 20  *C*: 1.0 | 0.82±0.19  *N*_PC_: 20  *C*: 1.0  *γ*: 0.01 | 0.80±0.16  *N*_PC_: 20  *C*: 100.0 | 3.19 |  | 3 |
| 0.81±0.14  *α*: 1.0  Hidden layer size: (128, 128, 64) | 0.91±0.14  Number of estimators: 64 | 0.81±0.18  Maximum depth: 5 | 0.84±0.10  *N*_PC_: 20  *C*: 1.0 | 0.82±0.17  *N*_PC_: 20  *C*: 10.0  *γ*: 0.001 | 0.79±0.16  *N*_PC_: 20  *C*: 10.0 | 3.23 |  | 4 |
| 0.60±0.08  *α*: 1.0  Hidden layer size: (128, 64, 32) | 0.71±0.11  Number of estimators: 256 | 0.58±0.09  Maximum depth: 20 | 0.63±0.09  *N*_PC_: 40  *C*: 1.0 | 0.65±0.10  *N*_PC_: 20  *C*: 100.0  *γ*: 0.001 | 0.64±0.10  *N*_PC_: 40  *C*: 1.0 | 3.75 |  | 5 |
| 0.82±0.13  *α*: 0.1  Hidden layer size: (128, 128, 64) | 0.89±0.17  Number of estimators: 512 | 0.76±0.13  Maximum depth: 50 | 0.78±0.15  *N*_PC_: 80  *C*: 0.1 | 0.77±0.15  *N*_PC_: 20  *C*: 100.0  *γ*: 0.001 | 0.79±0.14  *N*_PC_: 80  *C*: 1.0 | 5.11 |  | 6 |
| 0.81±0.16  *α*: 1.0  Hidden layer size: (128, 64, 32) | 0.86±0.18  Number of estimators: 512 | 0.78±0.15  Maximum depth: 5 | 0.82±0.19  *N*_PC_: 20  *C*: 10.0 | 0.83±0.17  *N*_PC_: 40  *C*: 100.0  *γ*: 0.001 | 0.82±0.18  *N*_PC_: 40  *C*: 10.0 | 3.34 |  | 7 |

# Measurements with Gas Flow Line

To compare the basic sensing response of the two MSS chips, we conducted gas sensing measurements with a gas flow line equipped with mass flow controllers (Fig. S15). Vapors of the solvents were injected to an MSS chip for 30 seconds, followed by nitrogen purge for 30 seconds. This cycle of sample gas injection and nitrogen purge was repeated four times for each solvent. The total gas flow rates of MFC1 and MFC2 were fixed at 100 standard cubic centimeters per minute (sccm). The flow rate of MFC1 for the sample gas injection was set at 20 sccm. Nitrogen was used as the carrier gas. The same devices were used for the readout as the free-hand measurement.


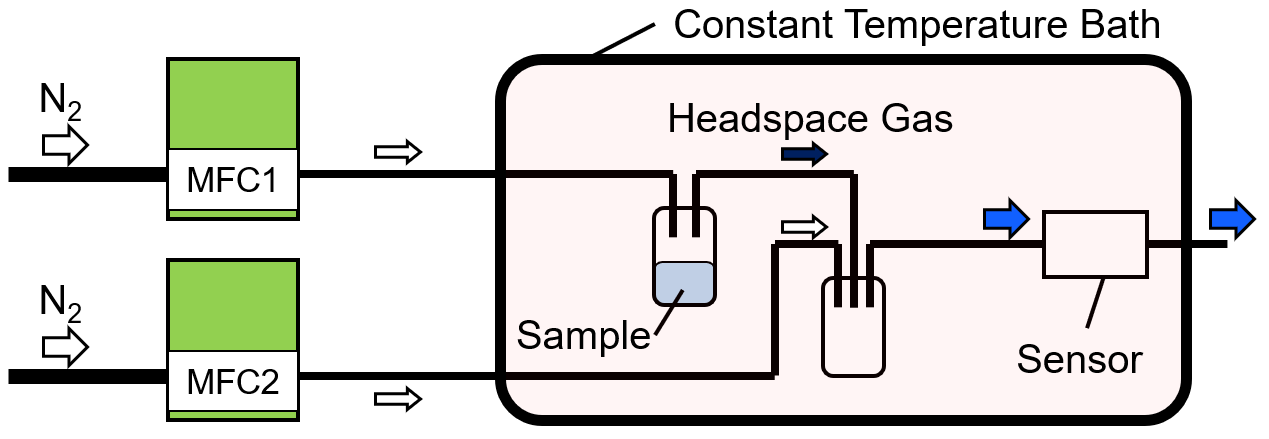


**Figure S15** Schematic illustration of the gas flow line based on MFCs.

## Sensing Responses of MSS Chip I to the Solvent Vapors Measured with MFC-Based Gas Flow Line


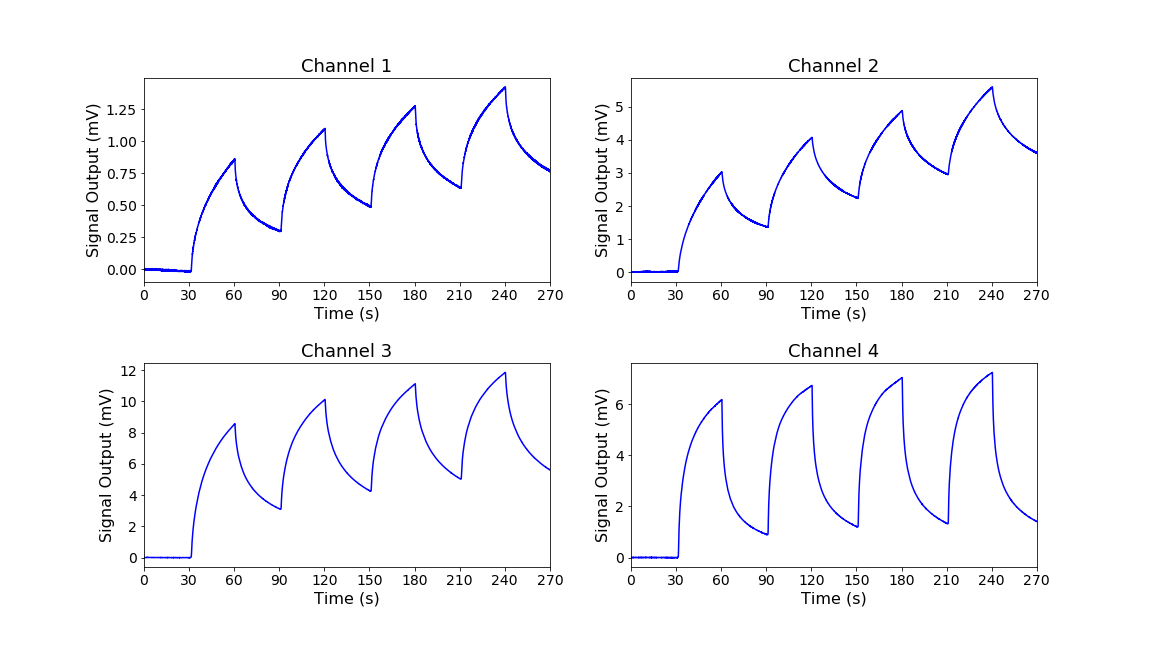


**Figure S16** Sensing responses of MSS Chip I to ethanol vapor with the rectangular gas flow sequence.


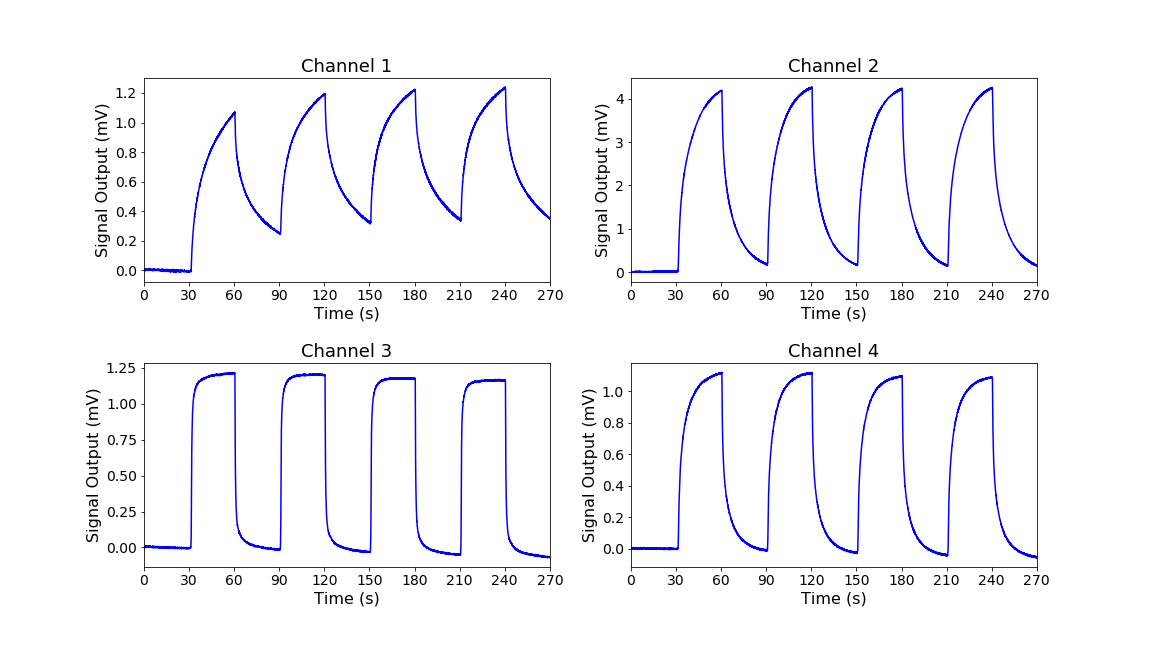


**Figure S17** Sensing responses of MSS chip I to water vapor with the rectangular gas flow sequence.


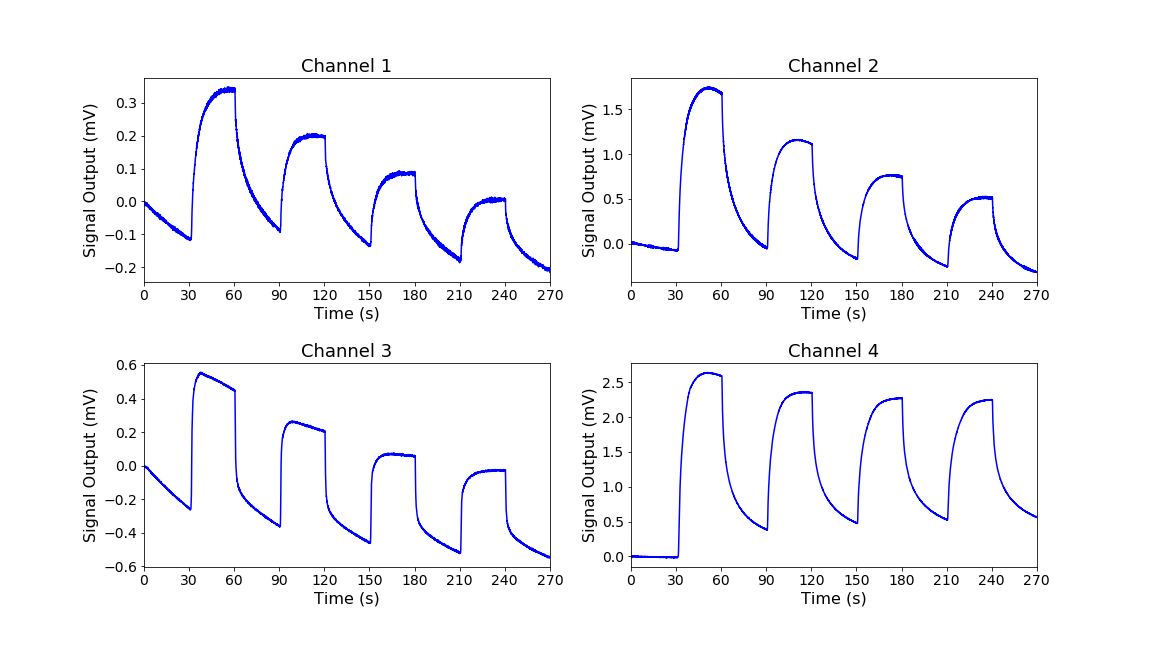


**Figure S18** Sensing responses of MSS chip I to heptane vapor with the rectangular gas flow sequence.


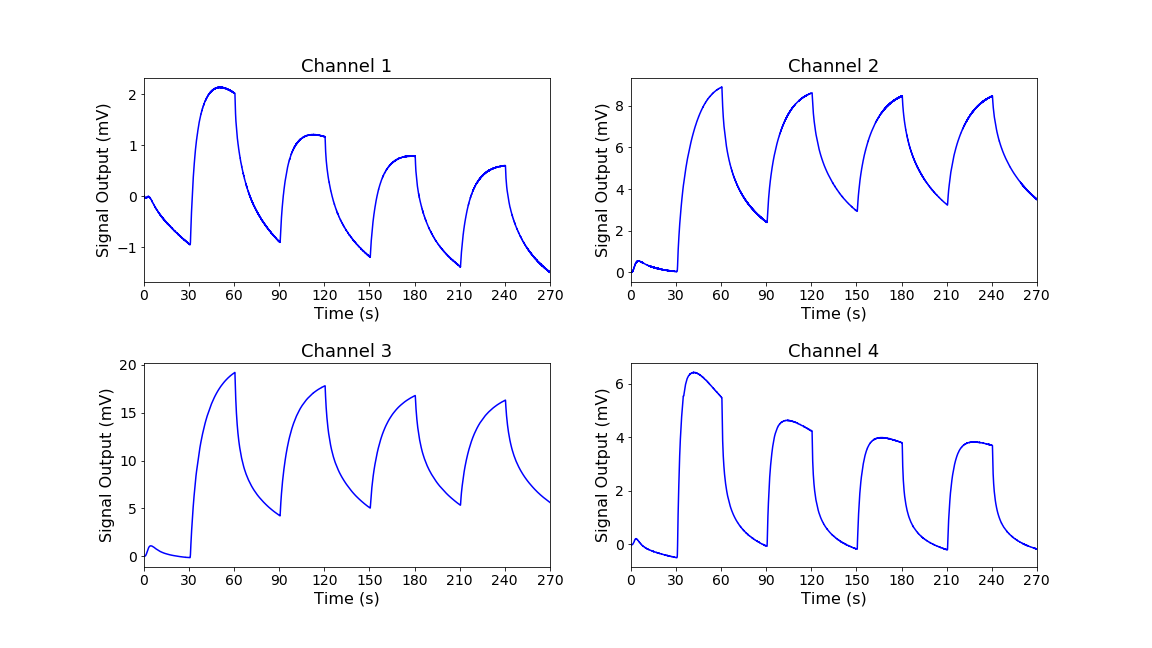


**Figure S19** Sensing responses of MSS chip I to ethyl acetate vapor with the rectangular gas flow sequence.

## Sensing Responses of MSS Chip II to the Solvent Vapors Measured with MFC-Based Gas Flow Line


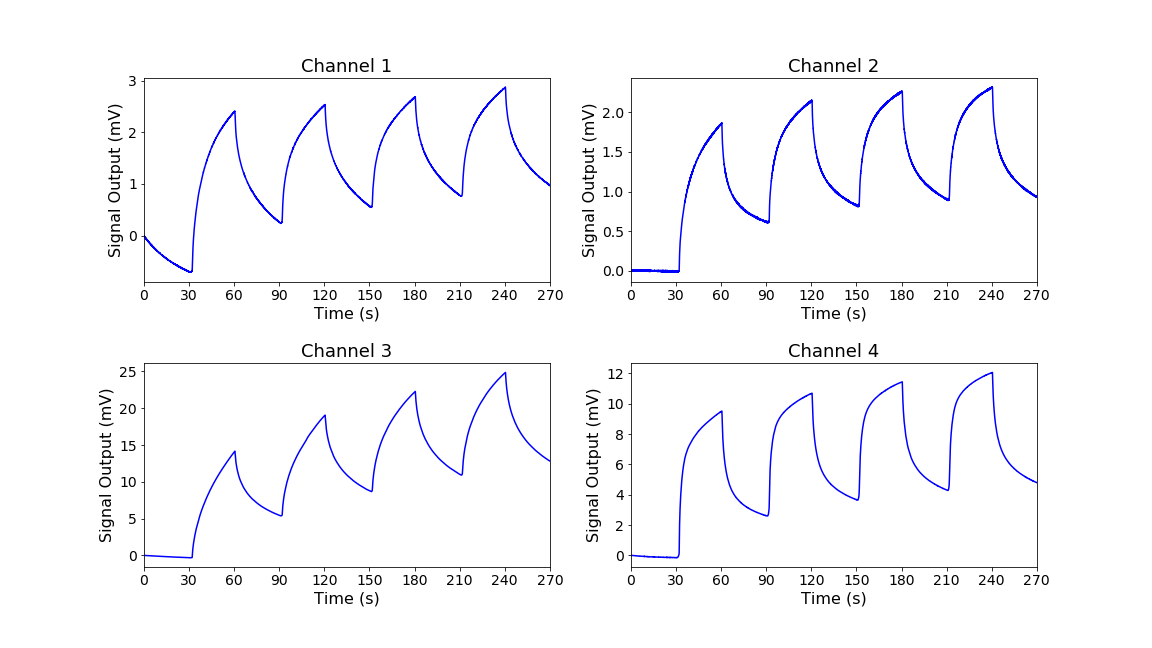


**Figure S20** Sensing responses of MSS chip II to ethanol vapor with the rectangular gas flow sequence.


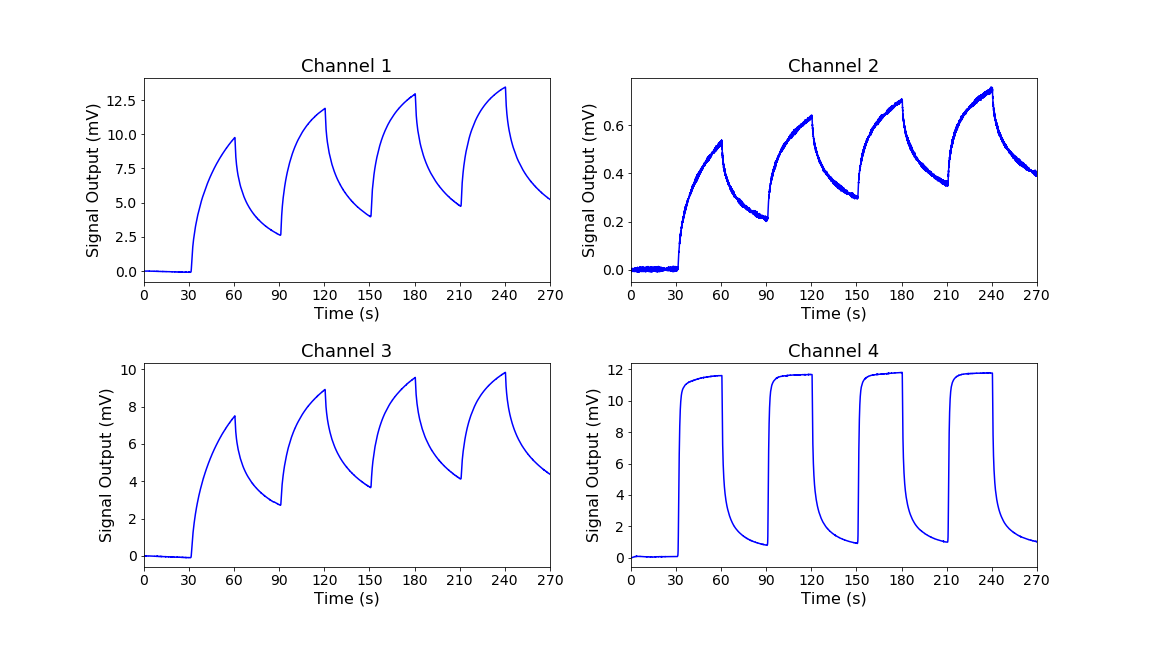


**Figure S21** Sensing responses of MSS chip II to water vapor with the rectangular gas flow sequence.


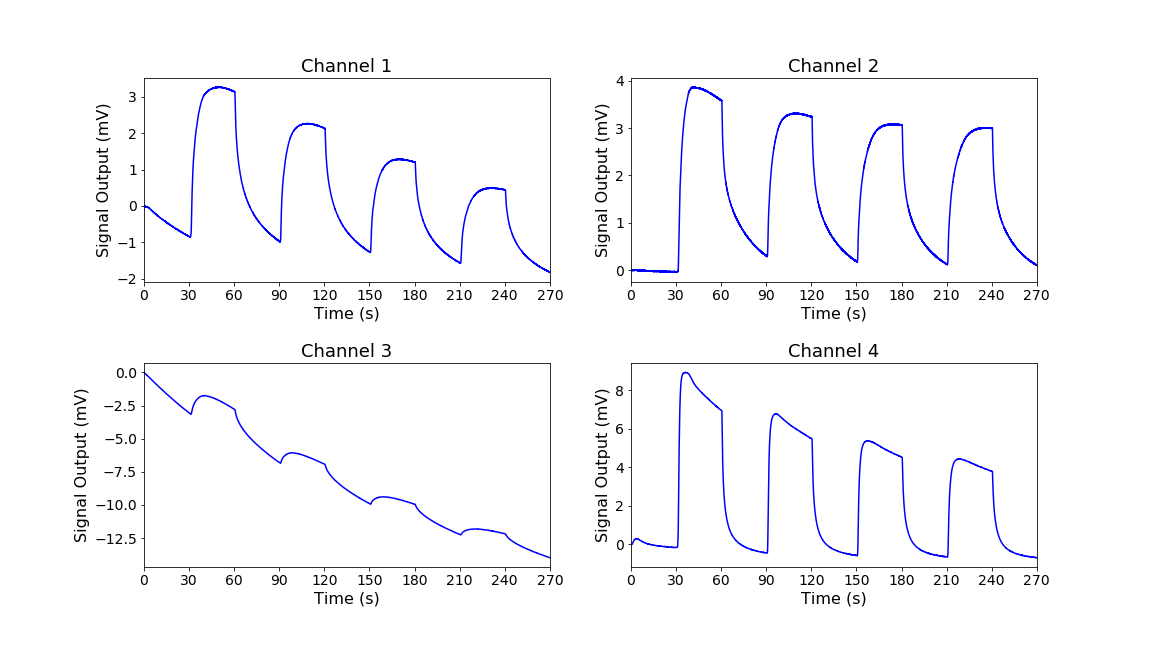


**Figure S22** Sensing responses of MSS chip II to heptane vapor with the rectangular gas flow sequence.


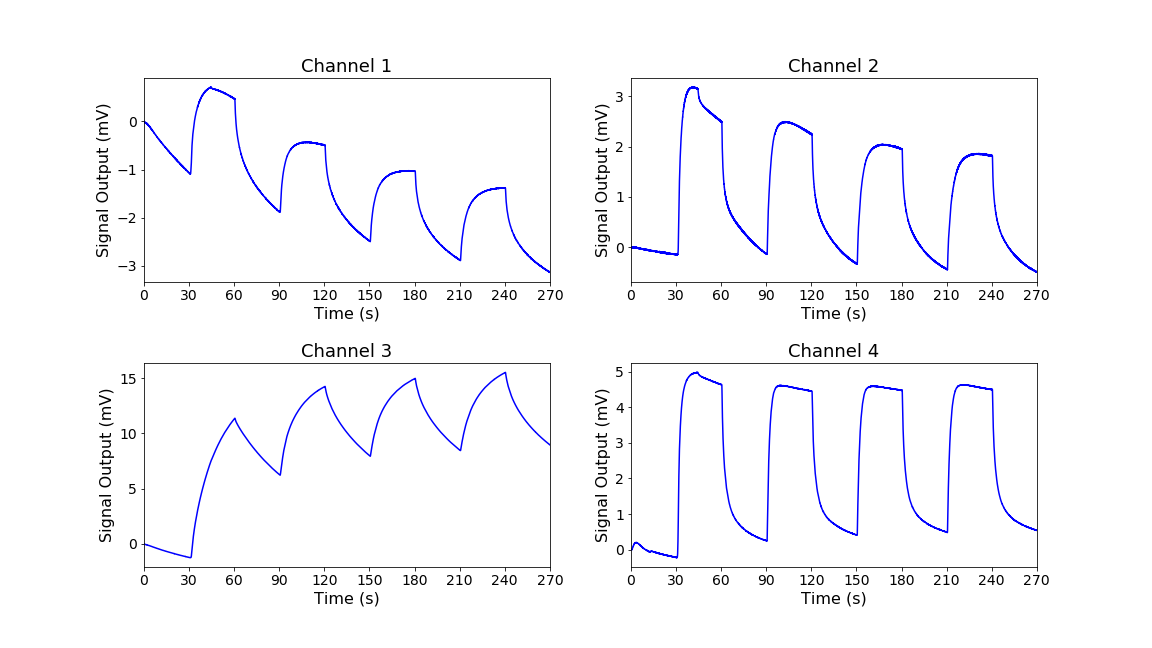


**Figure S23** Sensing responses of MSS chip II to ethyl acetate vapor with the rectangular gas flow sequence.

## Comparison between MSS Chip I and MSS Chip II

The intensities of a channel to the solvent vapors were calculated from the results of gas sensing measurements with the gas flow line (Figs. S16-S23). The intensities are summarized in Table S4. In terms of gas identification, it is preferable that each channel exhibits various selectivity to the vapors. To quantitatively evaluate the chemical selectivity of the chips, we calculated the variance in intensity for each channel; a channel which has a certain selectivity shows variable intensities to the vapors while a channel with a low selectivity shows a similar intensity to any vapor. The results are shown in the bottom of Table S4. The sums of the variances for MSS Chip I and MSS Chip II are 24.06 and 34.31, respectively. Thus, MSS Chip II shows more variable intensities to the vapors than MSS Chip I, indicating that MSS Chip II is more capable of discriminating the vapors than MSS Chip I.

Table S4. Intensities of channels of MSS Chip I and MSS Chip II. The normalized intensities are in the parenthesis.

|  | MSS Chip I | | | | MSS Chip II | | | |
| --- | --- | --- | --- | --- | --- | --- | --- | --- |
|  | **Ch. 1** | **Ch. 2** | **Ch. 3** | **Ch. 4** | **Ch. 1** | **Ch. 2** | **Ch. 3** | **Ch. 4** |
| Ethanol | 0.72  (0.36) | 2.29  (0.45) | 6.49  (0.60) | 5.85  (1.00) | 1.98  (0.23) | 1.40  (0.49) | 12.85  (1.00) | 7.49  (0.70) |
| Water | 0.88  (0.44) | 4.08  (0.80) | 1.22  (0.11) | 1.13  (0.19) | 8.42  (1.00) | 0.38  (0.13) | 5.55  (0.43) | 10.76  (1.00) |
| Heptane | 0.20  (0.10) | 0.79  (0.16) | 0.51  (0.05) | 1.70  (0.29) | 2.13  (0.25) | 2.88  (1.00) | 0.93  (0.07) | 4.47  (0.41) |
| Ethyl Acetate | 2.03  (1.00) | 5.07  (1.00) | 10.77  (1.00) | 3.88  (0.66) | 1.62  (0.19) | 2.28  (0.79) | 6.75  (0.53) | 3.98  (0.37) |
| Variance | 0.44 | 2.71 | 17.41 | 3.50 | 7.97 | 0.89 | 18.07 | 7.38 |
| Sum | 24.06 | | | | 34.31 | | | |

# Measurement of Spices

The odors of three dried spices and herbs—namely, rosemary, red chili pepper, and garlic—were measured with MSS Chip II through the free-hand measurement. Commercial spices from S&B Foods Inc. were used as samples. The spices were placed in glass beakers, and their vapors were measured.

## Sensing Responses of MSS Chip II to the Spices Through the Free-Hand Measurement


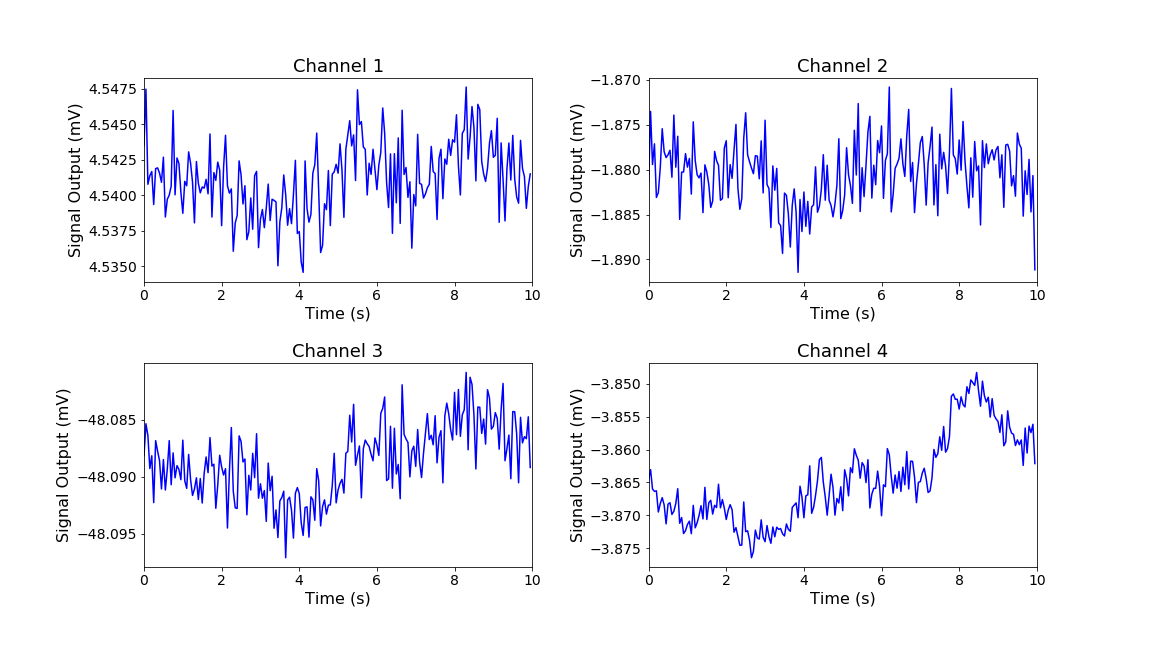


**Figure S24** Sensing responses of MSS chip II to rosemary with the free-hand measurement.


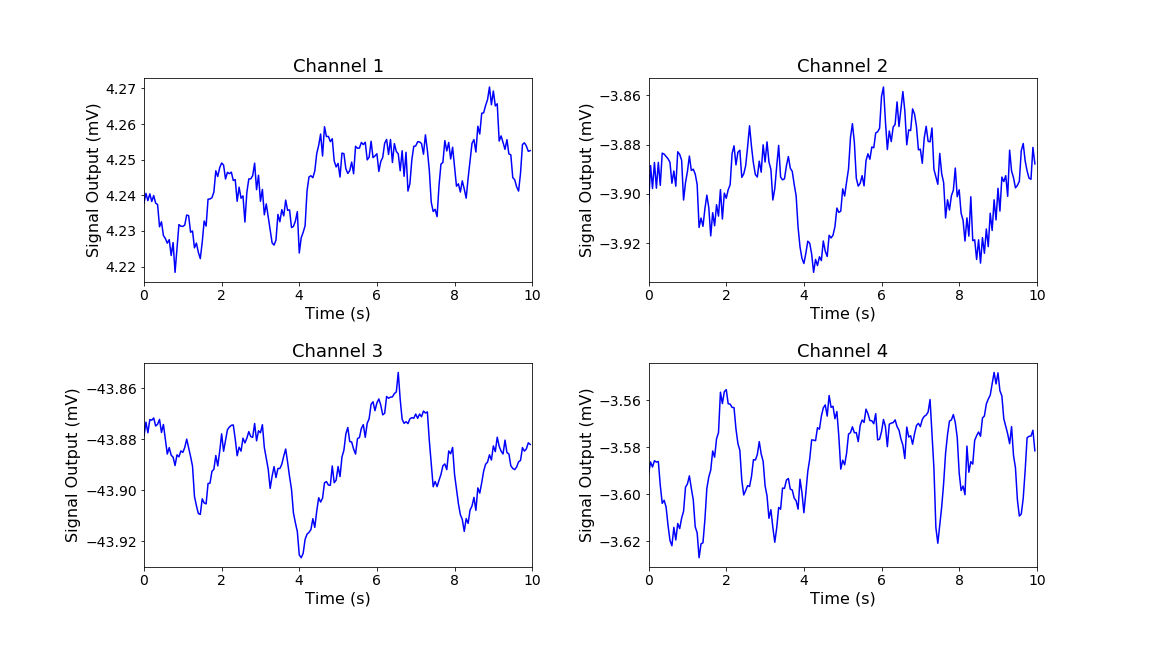


**Figure S25** Sensing responses of MSS chip II to red chili pepper with the free-hand measurement.


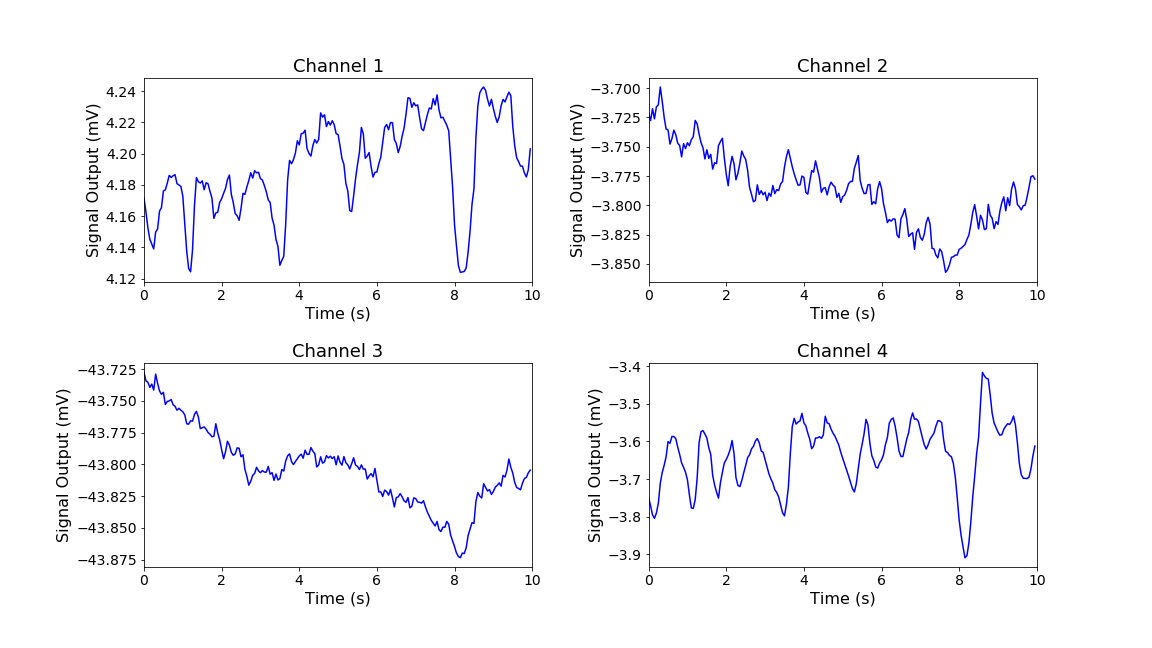


**Figure S26** Sensing responses of MSS chip II to garlic with the free-hand measurement.

# Development of Classification Models from Different Gas Input Patterns

The four solvent vapors were measured with two different gas input patterns: the m-sequence pseudorandom sequence and the rectangular sequence. The measurements were conducted with MSS Chip II by using the gas flow line (Fig. S13). The time-series data obtained with the two gas flow sequences were divided by 12 seconds. Based on the divided time-series data, the dataset of TFR was generated through the same procedure as in the case of the free-hand measurement. In this study, the frequency components of TFR at 0.083, 0.166, …, 0.833 Hz were used. The classification models were developed from the data obtained with the m-sequence pseudorandom sequence, followed by validation by using the data with the rectangular sequences as test data.

The results were compared with other analysis methods: AR models and FFT. The AR coefficients and the frequency components of the data were used as features for machine leaning. For the FFT-based analysis, the same frequency components were used as in the case of TFR-based analysis.

## M-sequence pseudorandom sequence

The binary sequence $[x_{0}, x_{1}, \cdots,x_{n}]$ was generated according to the following recurrence relation:

$$x_{k}=x_{k-q}+x_{k-p}$$

where $p>q>0$. Note that “$+$” represents the modulo-2 addition. In this study, we set $(p,q)=(5,1)$ and $n=47$. The MFC1 and MFC2 switched their gas flow rates at every 4 seconds. The flow rates of MFC1 and MFC2 were set at 20 and 80 sccm for $x_{n}=1$, while the flow rates of MFC1 and MFC2 were set at 0 and 100 sccm for $x_{n}=0$.

## Rectangular sequence

The sample gas and the carrier gas were alternatively injected to the MSS chip. For the sample gas injection, the flow rates of MFC1 and MFC2 were set at 20 and 80 sccm, respectively. The duration for sample gas injection and carrier gas purge was set at 6 seconds.

## AR models

If time-series data $y_{t}$ follows an AR model, $y_{t}$ can be described as the following form:

$$y_{t}=c+\sum_{j=1}^{p} \phi_{j}y_{t-j}+\epsilon_{t}$$

where $c$, $p$, $\phi_{j}$, $\epsilon_{t}$ are a constant, the maximum lag, the AR coefficients, and an error term, respectively. In this study, we set $p=20$ and developed AR models at each channel from the divided time-series data. From the AR models, $\phi_{j}$ were used as features for developing classification models.

# Identification of Three Solvent Vapors (Supplementary Video)

We performed the identification of three solvent vapors (i.e. water, ethanol, and isopropyl alcohol) through the free-hand measurement. The same experimental setup was used. We first measured the vapors of the three solvents with MSS Chip II for three times. Then, a gas identification model based on random forest was developed through 5×5 CV. Based on the developed model, the system predicts gas species from the sensing signals and shows the result every three seconds.
